# In-Depth Glycoproteomic Assay of Urinary Prostatic Acid Phosphatase

**DOI:** 10.1021/acsmeasuresciau.3c00055

**Published:** 2023-12-08

**Authors:** Wei Wang, Carmen R. de Nier, Manfred Wuhrer, Guinevere S.M. Lageveen-Kammeijer

**Affiliations:** † Leiden University Medical Center, Center for Proteomics and Metabolomics, Leiden 2300 RC, The Netherlands; ‡ University of Groningen, Groningen Research Institute of Pharmacy, Groningen 9713 AV, The Netherlands

**Keywords:** Diagnostic marker, Prognostic marker, Glycosylation, CE-MS, Prostate cancer, Prostatic acid phosphatase (PAP), Urinary assay

## Abstract

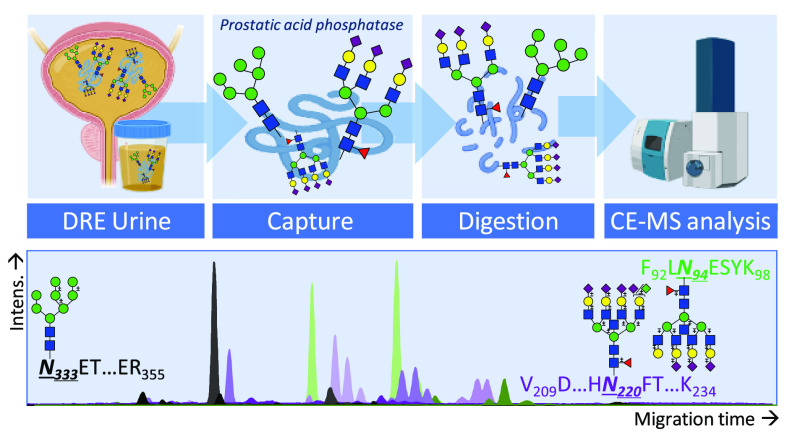

Prostate-specific antigen (PSA) is a well-known clinical biomarker in prostate cancer (PCa) diagnosis, but a better test is still needed, as the serum-level-based PSA quantification exhibits limited specificity and comes with poor predictive value. Prior to PSA, prostatic acid phosphatase (PAP) was used, but it was replaced by PSA because PSA improved the early detection of PCa. Upon revisiting PAP and its glycosylation specifically, it appears to be a promising new biomarker candidate. Namely, previous studies have indicated that PAP glycoforms differ between PCa and non-PCa individuals. However, an in-depth characterization of PAP glycosylation is still lacking. In this study, we established an in-depth glycoproteomic assay for urinary PAP by characterizing both the micro- and macroheterogeneity of the PAP glycoprofile. For this purpose, PAP samples were analyzed by capillary electrophoresis coupled to mass spectrometry after affinity purification from urine and proteolytic digestion. The developed urinary PAP assay was applied on a pooled DRE (digital rectal examination) urine sample from nine individuals. Three glycosylation sites were characterized, namely N_94_, N_220_, and N_333_, via *N*-glycopeptide analysis. Taking sialic acid linkage isomers into account, a total of 63, 27, and 4 *N*-glycan structures were identified, respectively. The presented PAP glycoproteomic assay will enable the determination of potential glycomic biomarkers for the early detection and prognosis of PCa in cohort studies.

## Introduction

Prostate cancer (PCa) is the second most common cancer in men. In 2020, approximately 1.4 million men were diagnosed with PCa and around 375 000 men died of it worldwide.^1^ In the majority of early stage PCa cases, no symptoms are observed.^2^ Early detection is essential to reduce the mortality of PCa and to improve the quality of patients’ life as well as treatability.^3^ As a biomarker for the early detection of PCa, the serum concentration of prostate-specific antigen (PSA) is clinically used; when elevated concentrations are found (>3 ng/mL (The Netherlands)), follow-up examinations are advised, such as a digital rectal examination (DRE), an ultrasonography, and an invasive prostate biopsy.^4^ Unfortunately, the PSA test is of limited value and tends to result in the overdiagnosis and overtreatment of patients,^5^ as it exhibits low specificity and poor predictive value for discriminating low-risk PCa from high-risk PCa; it is also unable to differentiate between PCa and other (benign) prostate-related diseases.^6−9^ Therefore, there is an urgent need for a more advanced non-invasive biomarker for the early detection of PCa.

Prior to the discovery of PSA, prostatic acid phosphatase (PAP) was used as a marker for the detection of PCa but was found to exhibit nonspecific expression in multiple organs.^10^ Similar to PSA, PAP is a glycoprotein mainly synthesized in prostate epithelial cells. Interestingly, its glycosylation, rather than its enzyme activity and concentration in serum, has been correlated to PCa in several studies, highlighting the potential of PAP glycosylation as a diagnostic biomarker of PCa. PAP is present in two different forms: cellular PAP (cPAP) and secreted PAP (sPAP). The two forms of PAP differ in glycosylation and have different isoelectric point (pI) values, which could be related to their sialylation levels.^11^ sPAP normally forms a dimer and shows phosphatase activity.^12^ A series of asparagine (N) to glutamine (Q) point mutations of PAP has proven that *N*-linked glycosylation is of critical importance for PAP stability (N_333_Q mutant) and its catalytic activity (N_94_Q single mutant, N_220_Q single mutant and N_94_Q and N_220_Q double mutant).^13^ It has been observed that the mutation of the *N*-linked glycosylation site N_333_, which is closest to the His_12_ in the active site of PAP, had a greater effect on protein activity and stability than the mutation of sites N_94_ and N_220_, which are further away.

Various *N*-glycan structures on PAP derived from human seminal plasma were first elucidated by ^1^H nuclear magnetic resonance spectroscopy in 1987.^14^ In this study, one oligomannosidic *N*-glycan and six complex-type *N*-glycans were reported. The complex-type *N*-glycans were fucosylated and partially sialylated, of which four were diantennary structures and two were triantennary structures. Later, Jakob et al. studied the crystal structure of seminal PAP and revealed that two *N*-glycosylation sites (N_94_ and N_333_) are actually occupied with oligomannosidic *N*-glycans and that only site N_220_ is occupied with complex-type *N*-glycans.^15^ In 1997, Yoshida et al. reported altered *N*-linked sugar chains of PAP during oncogenesis in the prostate tissue by means of lectin affinity chromatography. In this study, low levels of oligomannosidic *N*-glycans and hybrid-type *N*-glycans with core fucose were observed in PCa compared to benign prostatic hyperplasia (BPH) as well as high levels of hybrid-type *N*-glycans without core fucose.^16^ With the rapid development of mass spectrometry (MS), a more in-depth analysis of glycosylation became available. White et al. studied the glycomic profile of PAP derived from seminal plasma and reported 21 *N*-glycan structures (*N*-glycan release) by matrix-assisted laser desorption/ionization (MALDI) coupled to time-of-flight mass spectrometry (TOF-MS) in 2009.^17^ To identify the site occupancy, glycopeptide analysis was performed, and similar findings as those observed in previous studies were found except for the *N*-glycosylation site N_94_, which appeared to be occupied with complex-type *N*-glycans rather than oligomannosidic ones, as was reported by Jakob et al.^15^ On the basis of the released *N*-glycan analysis of PAP, lower levels of fucosylated di- and tetraantennary as well as oligomannosidic *N*-glycans were found, while an increase of smaller monoantennary *N*-glycans was found for the pool of PCa patients (*n* = 92) compared to normal control (*n* = 65) and BPH (*n* = 59) pools.^17^ Another MS study in 2013 demonstrated similar glycosylation features for the *N*-glycosylation sites N_94_ and N_220_ (occupied with complex-type *N*-glycans) on PAP derived from DRE urine but also an increase of bisected structures. Interestingly, in this study, the *N*-glycosylation site N_333_ was not only occupied with oligomannosidic *N*-glycans but also with complex-type *N*-glycans.^18^ Additionally, by using sialic acid- and fucose-binding lectins, a significant decrease in captured PAP was observed in the DRE urine pool of aggressive PCa (*n* = 10) compared to indolent PCa (*n* = 10).^18^ A more recent study by Sugár et al. in 2021 analyzed cancerous (*n* = 49) and healthy (*n* = 46) prostate tissues from biopsies and characterized the differences in their glycosylation using an untargeted bottom-up approach. It was revealed that there was an increase in fucosylation and a decrease in sialylation for the *N*-glycosylation site N_94_ of PAP.^19^

While these MS studies explored PAP glycosylation to a certain extent, limitations were encountered regarding the coverage of site N_220_ as well as the coverage of minor glycoforms. Additionally, for the glycoproteomic studies, missed proteolytic cleavages for all three glycosylation sites resulted in complex data sets.^17,18^ Moreover, no distinction could be made between isomeric sialylated species despite the indications of the profoundly different roles of those isomers in cancer in general: α2,6-linked isomers have been implicated in blocking galectin binding and enhancing tumor cell survival, while α2,3-linked isomers are considered as a hallmark of malignant types of cancers.^20−23^ Therefore, to gain further insight into PAP glycosylation and its biomarker potential in relation to PCa, an in-depth characterization of the PAP *N*-glycome is of critical importance.

In this study, we report an in-depth urinary PAP glycoproteomic assay (uPGA). The assay was successfully applied on a pooled urine sample collected from nine males after DRE. The developed method is a stepping stone for defining the biomarker potential of PAP glycosylation for patient stratification in relation to PCa.

## Materials and Methods

### Chemicals

See the Supporting Information (SI), section S-1.1.

### Samples

Urine samples were collected from healthy female volunteers at the Leiden University Medical Center. One female urine pool (FUP) was formed by pooling all collected urine samples together. Before pooling, the urine samples were centrifuged for 5 min at 2500*g* at 5 °C, and the pH was measured. The obtained FUP was aliquoted and stored at −20 °C. Prior to use, the FUP was thawed to room temperature (rt); then, it was centrifugated for 5 min at 500*g* to fasten the precipitation of the urinary sediments. The DRE urine pool was prepared by Roche (Penzberg, Germany). The DRE urine samples were collected according to the appropriate standard operating procedures approved by local ethic committees (for sample information, see the SI, Table S-1). Samples were stored at below −70 °C until analysis. Repeated freeze–thawing was avoided. First, all nine individual samples were diluted by a factor of 1000 or 10 000 to fit in with the measurement range of the PAP concentration assay (developed by Roche). Then, 1 mL from each sample was used to form the urine pool (Table S-1).

### Anti-PAP Beads

Two biotinylated anti-PAP antibodies, anti-PAP I (1.39 mg/mL) and anti-PAP II (1.08 mg/mL), were provided by Roche. The antibodies were mouse monoclonal antibodies against PAP antigen using hybridoma technology (generated in-house by Roche).^24^ The two different antibodies were separately coupled to high-capacity streptavidin agarose resins (HCS beads). The HCS beads were prewashed four times with 1× phosphate-buffered saline (PBS). Then, 500 μL of the drained beads was added to 5 mg of the anti-PAP antibodies. The coupling of the antibodies with the beads was achieved by incubating them overnight (ON) on a roller shaker (Roller 10 digital, IKA Laboratory Technology, Staufen, Germany) at 4 °C. The coupled beads were washed twice with 1× PBS to remove any unbound antibodies. To remove unspecific bindings, the beads were incubated for 3 min with 100 mM formic acid (FA), followed by a wash with 1× PBS to adjust the pH to 7. The supernatant was removed after centrifugation (2 min at 100*g*). A 50% bead suspension was made by resuspending the beads in 1× PBS with 0.02% NaN_3_ (*v/v*). Prior to usage, the coupled anti-PAP beads were stored at 4 °C. The coupling efficiency was determined by comparing the same volume prior to (approximately 9 μg of antibodies) and after coupling the antibodies by sodium dodecyl–sulfate polyacrylamide gel electrophoresis (SDS-PAGE) analysis (SI, section S-1.2).

### PAP Capture

To develop the PAP capture protocol, 250 μL of 5× PBS spiked with 1.5 μg of the PAP standard was added to 1 mL of FUP. Male urine was considered unsuitable to use during the development of the PAP capturing procedure, as it contains PAP. In contrast, FUP contains no PAP, and the matrix is the closest to that of male urine samples; hence, it was chosen for use for protocol development. By spiking the PAP standard to the FUP, we were able to mimic male urine samples. Different amounts of HCS beads and different densities of bead suspension (2, 4, and 10 μL of 25% bead suspension and 2 and 5 μL of 50% bead suspension) were evaluated to determine the optimal affinity purification procedure, and a series of FA concentrations (5, 10, 25, 50, 100, and 150 mM) were evaluated for the elution buffer. All experiments were performed in duplicate. In all cases, the yield was evaluated by SDS-PAGE.

The optimized protocol used 2 μL of 50% anti-PAP I bead suspension, which was added to a mixture containing 1 mL of FUP and 250 μL of 5× PBS with 1.5 μg of the PAP standard or 1 mL of diluted, pooled DRE urine (∼3.2 μg/mL) and 250 μL of 5× PBS. Incubation was performed ON at 4 °C by constantly rotating the tubes horizontally. Afterward, the beads were washed with 1× PBS and transferred to a 96-well polypropylene filter plate with a 10 μm polyethylene (PE) frit (Orochem, Naperville, Illinois, U.S.). By use of a vacuum manifold (Merck Millipore, Darmstadt, Germany), the beads were sequentially washed once with 600 μL of 1× PBS and twice with 600 μL of 50 mM ammonium bicarbonate. For elution, 200 μL of 150 mM FA was added; then, the samples were placed on a plate shaker for 5 min (1000 rpm, Heidolph Titramax 100 platform shaker, Heidolph, Schwabach, Germany). The eluate was collected in a low protein binding flat bottom plate (polypropylene plate, Greiner Bio-One, Kremsmünster, Austria) by centrifugation (2 min, 100*g*). The samples were then dried by a vacuum concentrator at 45 °C for 2 h.

### Tryptic Digestion

Various conditions and enzymes were explored for the most optimal digestion of PAP, and different reduction and alkylation strategies were compared. Briefly, four different enzymes with different properties were explored (*N*-tosyl-l-phenylalanine chloromethyl ketone (TPCK)-treated trypsin from Sigma-Aldrich (Steinheim, Germany), trypsin gold, trypsin platinum, and sequence grade modified (SGM) trypsin from Promega (Madison, Wisconsin, U.S.A.) investigated) as well as different enzyme:protein ratios (1:5, 1:10, 1:20, and 1:50). For all digestion experiments, 200 ng of the PAP standard was used. In the case of dried samples, reconstitution was performed in 5 μL of 25 mM ammonium bicarbonate.

The final procedure used the following conditions: reduction was performed by adding 1 μL of 12 mM DL-dithiothreitol (DTT) to the samples, followed by a heating step at 60 °C for 30 min. Afterward, the samples were alkylated by adding 1 μL of 48 mM iodoacetamide (IAA) and left in the dark for 30 min at rt. Next, 1 μL of 48 mM DTT was added, and the samples were incubated in light for 20 min at rt. Then, the samples were proteolytically digested with SGM trypsin by adding 1 μL of 200 ng/μL SGM trypsin ON. Digestion was quenched by adding 1 μL of 5% FA to the samples.

### Capillary Electrophoresis–Mass Spectrometry (CE-MS/MS)

The identification of PAP glycopeptides was performed on a bare-fused silica capillary (inner diameter = 30 μm, total length = 91 cm) using a CESI 8000 system (SCIEX, Brea, California, U.S.) coupled to a UHR-QqTOF-MS instrument (Impact; Bruker, Bremen, Germany). Prior to injection, 4 μL of tryptic PAP glycopeptides (20 ng/μL) was mixed with 2 μL of leading electrolyte (LE, 1200 mM ammonium acetate at pH = 3.2). The capillary voltage was set at 20 kV, and the capillary temperature was set at 15 °C. Injection was executed by applying 25 psi of pressure for 24 s, corresponding to 13.5% of the capillary volume (87 nL). All experiments were performed in positive ionization mode, and intensities were enhanced by the use of dopant-enriched nitrogen (DEN) gas (nanoBooster, Bruker) with acetonitrile as a dopant at 0.2 bar.^25^ The temperature and flow rate of the drying gas were set at 150 °C and 1.2 L/min, respectively. Fragmentation data were acquired on the three most abundant precursor ions between a mass-to-charge ratio (*m*/*z*) of 150 and 2500 with a 1 Hz spectral acquisition frequency and a minimum intensity of 3757. Depending on the *m*/*z* values, the precursor ions were isolated with a width of 8–15 Th. The collision energies were set as a linear curve in an *m*/*z*-dependent manner, ranging from 20 eV at an *m*/*z* of 500 to 70 eV at an *m*/*z* of 2000 for all charge states (1–5), applying a basic stepping mode with collision energies of 100% (80% of the time) to 70% (20% of the time). A mass exclusion list was applied during fragmentation to avoid the influence of co-captured proteins (see the SI, Table S-2).

### Liquid Chromatography–Tandem Mass Spectrometry (LC-MS/MS)

See the SI, sections S-1.3 and S-1.4.

### Assay Assessment

The limit of detection (LOD) of the established assay was determined by spiking the PAP standard (0, 20, 50, 100, and 200 ng) into one mL of FUP, followed by executing the complete protocol including PAP capturing, tryptic digestion, and CE-MS analysis. The inter- and intraday validation of the assay was assessed by spiking 200 ng of the PAP standard to 1 mL of FUP over three separate days with four replicates per day and executing the complete protocol.

### Data Analysis

Both the LC-MS(/MS) (see the SI, section S-1.3) and CE-MS(/MS) data were analyzed with the Compass DataAnalysis 5.0 SR1 (x64) software (Build 203.2.3586, Bruker). With DataAnalysis, all of the MS and tandem MS data were manually screened for glycopeptides based on exact *m*/*z* values, migration/elution order, and relative abundance of the analytes. Extracted ion electropherograms (EIEs)/extracted ion chromatographs (EICs) were acquired with the first three isotopes of the singly, doubly, triply, and quadruply charged analytes ranging from 200 to 2500 Da using a width of ±*m*/*z* 0.02 Da. The tandem MS data acquired from LC-MS (Orbitrap; see the SI, section S-1.4) were analyzed with the Xcalibur software (Thermo Fisher Scientific, 2.2 SP1.48) for the structural elucidation of the glycopeptides. Additionally, Byonic (v4.6.1, Protein Metrics, Inc.) searching was performed using a homo sapien protein database acquired from UniProt.

## Results and Discussion

**Figure 1:**
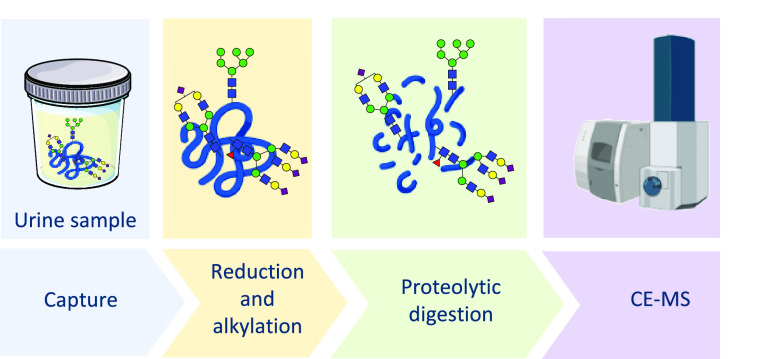
In-depth urinary PAP glycoproteomic workflow. PAP was enriched from DRE urine by immunoaffinity capturing overnight. After capturing, PAP was proteolytically digested and directly subjected to CE-MS measurements. Three glycosylation sites were identified and characterized in an in-depth manner.

**Figure 2:**
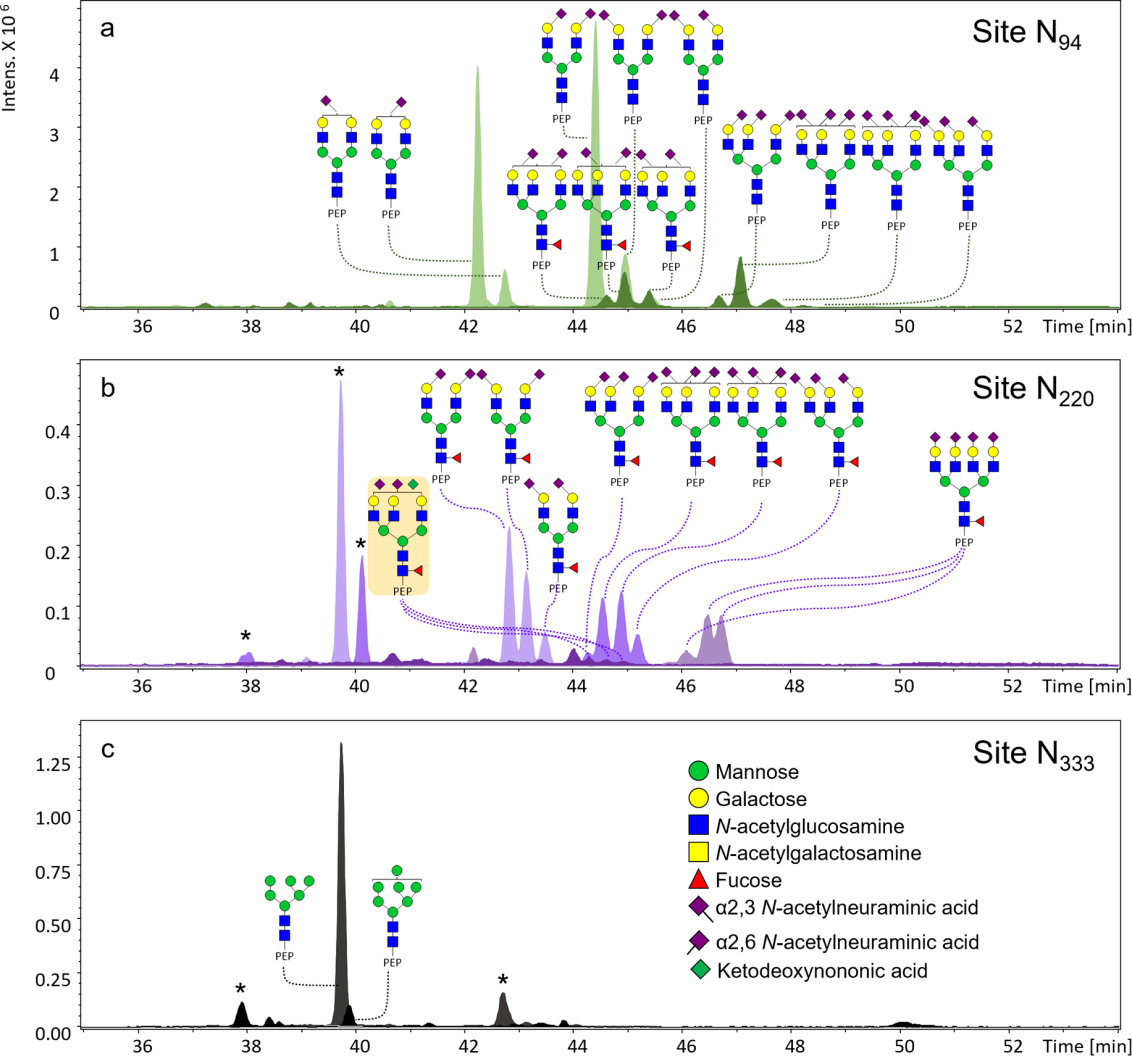
Extracted ion electropherograms (EIEs) of the most abundant glycans on DRE urinary PAP on (a) site N_94_, (b) site N_220_ and (c) site N_333_. The glycan structure with a yellow background color in part (b) highlights the ketodeoxynononic acid (Kdn)-containing glycan identified on PAP. Three isomers of the Kdn-containing glycan were observed. PEP: peptide backbone. Asterisk indicates non-glycopeptide analytes. Peptide backbone of site N_94_ is FLN_94_ESYK. Peptide backbone of site N_220_ is VYDPLYCESVHN_220_FTLPSWATEDTMTK. Peptide backbone of site N_333_ is N_333_ETQHEPYPLMLPGCSPSCPLER.

A urinary PAP glycoproteomic assay (uPGA) was established (Figure 1). To begin, PAP was captured from 1 mL of urine; then, it was proteolytically digested and directly measured with CE-MS. The established uPGA resolved all three glycosylation sites on the DRE urinary PAP and revealed multiple glycoforms per site, including the distinction of sialic acid linkage-specific isomers on a glycopeptide level. Signature glycosylation features were identified (e.g., sialylation, fucosylation, and branching) and characterized, and their relative abundance was determined in an in-depth manner (relative abundance <0.1%; Figures 2 and S-1 and Tables S-3 and S-4).

### PAP Capturing

For optimization of the capture procedure, several parameters were tested. First, a successful coupling of the two anti-PAP antibodies was assessed by SDS-PAGE (Figure S-2). Then, the optimal elution buffer was determined. For this purpose, different FA concentrations (5, 10, 25, 50, 100, and 150 mM) were investigated by SDS-PAGE (*n* = 2; Figure S-3). A higher concentration of FA resulted in a higher yield for the anti-PAP I beads, with 150 mM FA being the most optimal elution buffer. As for the anti-PAP II beads, with the exception of 5 mM FA, the yields with different FA concentrations were comparable. Thus, 150 and 50 mM FA was chosen as elution buffers for the anti-PAP I and anti-PAP II beads, respectively. Next, the bead volume was evaluated in combination with different quantities of anti-PAP beads (2, 4, and 10 μL of 25% bead suspension and 2 and 5 μL of 50% bead suspension, *n* = 2; Figure S-4). The highest capturing efficiency with the lowest variation was found with 2 μL of 50% bead suspension for both anti-PAP I and anti-PAP II. The capture efficiencies were determined to be 75% ± 1% and 73% ± 2% for anti-PAP I and anti-PAP II, respectively. To investigate whether any capturing bias existed for the two different antibodies in regard to the glycosylation profile, the glycomic profiles were compared before and after capturing by CE-MS (*n* = 3; Figure S-5). No significant differences were observed between the two anti-PAP beads. On the basis of the yields, anti-PAP I was selected for further experiments.

### Proteolytic Cleavage

Initially, 1.5 μg of the PAP standard was used for the digestion with TPCK-treated trypsin. Then, to optimize the digestion, subsequent experiments started with a decreased amount of the PAP standard (200 ng). A total of four different peptide backbones were found for the *N*-glycosylation site N_94_, including zero, one, or two missed cleavages. Two peptide variants were found for sites N_220_ and N_333_ with zero or one missed cleavage (Table S-5). The observed missed cleavages on the peptide backbones are probably due to multiple cleavage sites being in close position to each other, such as LGEYI**RKR**Y**RK**FLN_94_ESY**K**HE (cleavage sites are bolded). Another cause might be the proximity of the cleavage sites to the *N*-glycosylation site, meaning the protease could be hampered by the large glycan moiety.^17^ Similarly, in a previous study by White et al., the PAP glycosylation was analyzed after digestion with trypsin or chymotrypsin, and both proteases resulted in missed cleavages.^17^

As the high variation in peptide backbones increased the data complexity, different types of trypsin and different enzyme:protein ratios were investigated to potentially improve the digestion efficiency and specificity. For this purpose, three different trypsin preparations with higher specificities and stabilities compared to the conventional TPCK-treated trypsin were selected. Whereas SGM trypsin has been developed to be highly active with a high specificity and stability compared to TPCK trypsin, trypsin gold has been designed to have a maximum digestion specificity in combination with a high resistance for autolytic cleavage. The final selected enzyme, trypsin platinum, is free of any detectable nonspecific proteolytic activity and has maximal autoproteolytic resistance, in addition to a high digestion efficiency. For all four types of trypsin, various ratios of the enzyme:protein were investigated (1:5, 1:10, 1:20, and 1:50). The *N*-glycosylation site N_220_ was not detected using TPCK-treated trypsin, which was therefore excluded from further experiments. Among all of the different ratios, the ratio of 1:5 presented the highest number of PAP glycopeptides independently of the selected enzyme (Table S-6). A more in-depth comparison was performed with this ratio (Figure S-6). For the *N*-glycosylation site N_94_, four peptide backbones were observed for all enzymes (Table S-6). The most abundant peptide backbone was found to be FLN_94_ESYKHEQVYIR (one missed cleavage) with a relative abundance of 63%, 71%, and 60% for SGM trypsin, trypsin gold, and trypsin platinum, respectively. The highest analyte area was observed with trypsin platinum, followed by trypsin gold and SGM trypsin, indicating that trypsin platinum has highest digestion efficiency. SGM trypsin showed the smallest variation (with an average relative standard deviation (RSD) of 8.0%), indicating higher robustness and higher repeatability, followed by trypsin platinum (average RSD of 9%). The largest variation was found for trypsin gold with an average RSD of 21%. The obtained relative abundance values from all three types of trypsin were comparable with average RSDs of 8%, 6%, and 8% for SGM trypsin, trypsin gold, and trypsin platinum, respectively. For the *N*-glycosylation site N_220_, SGM trypsin outperformed the two other enzymes with a subsequently higher area for most of the analytes and a small variation between replicates. Regarding the analytes area, the average RSDs were 13%, 34%, and 41% for SGM trypsin, trypsin gold, and trypsin platinum, respectively. However, among the different trypsin types, a visible difference in the relative abundance was found. Compared to SGM trypsin, the glycomic profile acquired from trypsin platinum presented a higher abundancy for diantennary *N*-glycans, while lower abundance values were found for tri- and tetraantennary *N*-glycans. This indicates that trypsin platinum and trypsin gold were likely hampered in their access to the *N*-glycosylation site N_220_, especially when occupied with larger *N*-glycans, such as tri- and tetraantennary *N*-glycans. Regarding their relative area, average RSDs of 7%, 15%, and 27% for SGM trypsin, trypsin gold, and trypsin platinum were found, respectively. For the *N*-glycosylation site N_333_, the highest number of *N*-glycans and the highest area were found using trypsin platinum, although SGM trypsin showed the smallest variation with an average RSD of 3% in analytes area. The average RSDs of trypsin gold and trypsin platinum were 36% and 20% respectively, which are roughly ten times higher than that of SGM trypsin, implying the digestion efficiencies of trypsin gold and trypsin platinum have poor repeatability. For the two *N*-glycans detected by all types of trypsin, the relative abundance values were comparable.

Considering the coverage for all three *N*-glycosylation sites, SGM trypsin was considered for further optimization, especially as SGM trypsin could potentially provide more information in the case of PAP samples with low concentrations. Namely, *N*-glycans attached to *N*-glycosylation site N_220_ have lower recoveries than the other two glycosylation sites and, more importantly, *N*-glycans occupying this site possess important *N*-glycomic features, such as fucosylation, sialylation, and a higher level of branching, which could be interesting for cancer biomarker discovery studies (Figure S-7).^26−29^ Furthermore, PAP has three disulfide bonds. To facilitate digestion, the disulfide bonds are generally reduced with a reducing reagent. Subsequently, an alkylation step should be accomplished to prevent the broken disulfide bonds from reforming. This process can be accomplished with different strategies. The most commonly used reducing reagent is DTT, and alkylation is usually performed by IAA. To optimize the digestion of PAP, the method of using DTT as a thiol reducing agent, together with using IAA for alkylation, was examined. Additionally, tris(2-carboxyethyl)phosphine (TCEP) was also assessed for PAP reduction in combination with chloroacetamide (CAA) for alkylation. However, the TCEP-treated samples did not show better digestion, and extra sample clean-up was required prior to MS analysis. Upon reduction and alkylation, RapiGest SF surfactant was also evaluated for PAP digestion, as it is commonly used to enhance the enzymatic digestion of proteins. It is a mild denaturant that helps to unfold proteins and expose the proteolytic sites to enzymatic cleavage sites. However, in the case of PAP digestion, no significant improvements in the digestion efficiency or specificity were observed (data not shown).

### Assay Assessment and Data Analysis

To explore the glycosylation of PAP, tryptic PAP was measured with both LC-MS and CE-MS to find the most suitable analytical platform. The data acquired from LC-MS showed no separation on sialic acid linkage-specific isomers for sites N_94_ and N_220_, while the isomer separation was achieved by CE-MS. A high level of sialylation was observed on the PAP *N*-glycans in our study (Figure S-7), and the differentiation of sialylation isomers was considered to be a critical requirement, as this important glycosylation feature is a known hallmark of cancer. Namely, differently linked sialic acids may play different roles in different types of cancer.^30−34^ Thus, CE-MS was chosen for its excellent glycoform separation power.

**Figure 3:**
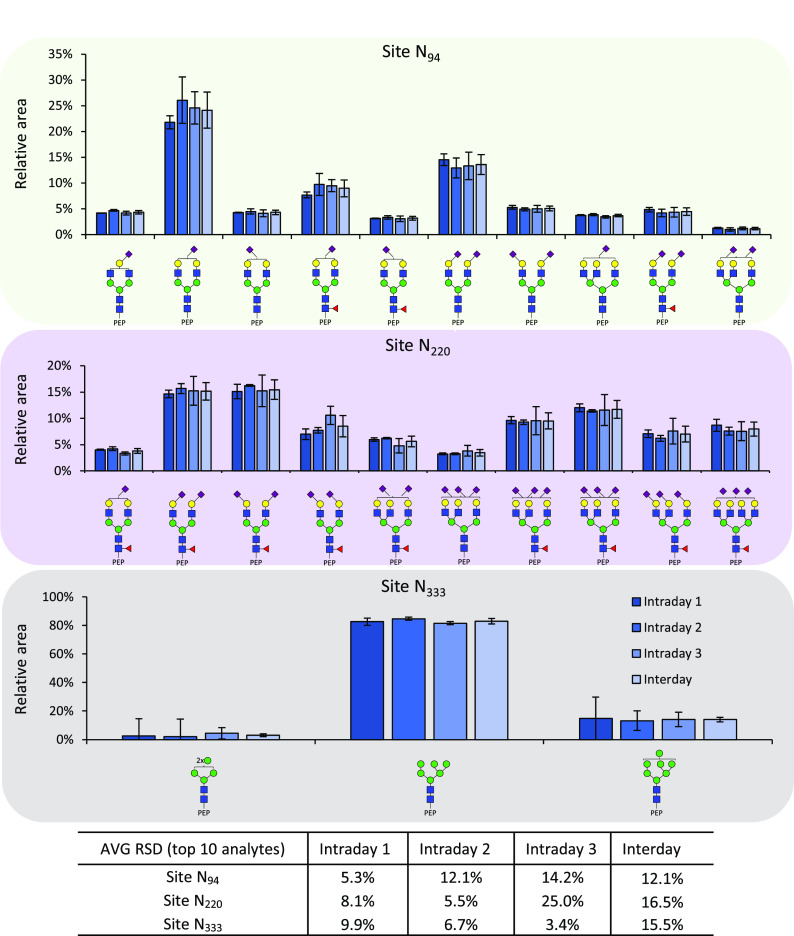
Intra- and interday validation of the developed prostatic acid phosphatase (PAP) glycoproteomic assay, showing the relative abundance of the 10 most abundant glycopeptides per glycosylation sites. The seminal plasma PAP standard was spiked into a female urine pool (FUP), and an in-solution digestion was performed and used to test the repeatability (intraday, *n* = 4) and intermediate precision (interday, *n* = 12) of the PAP glycoproteomic assay by CE-MS. For site N_333_, the average relative standard deviation (AVG RSD) shows the top three analytes.

**Figure 4:**
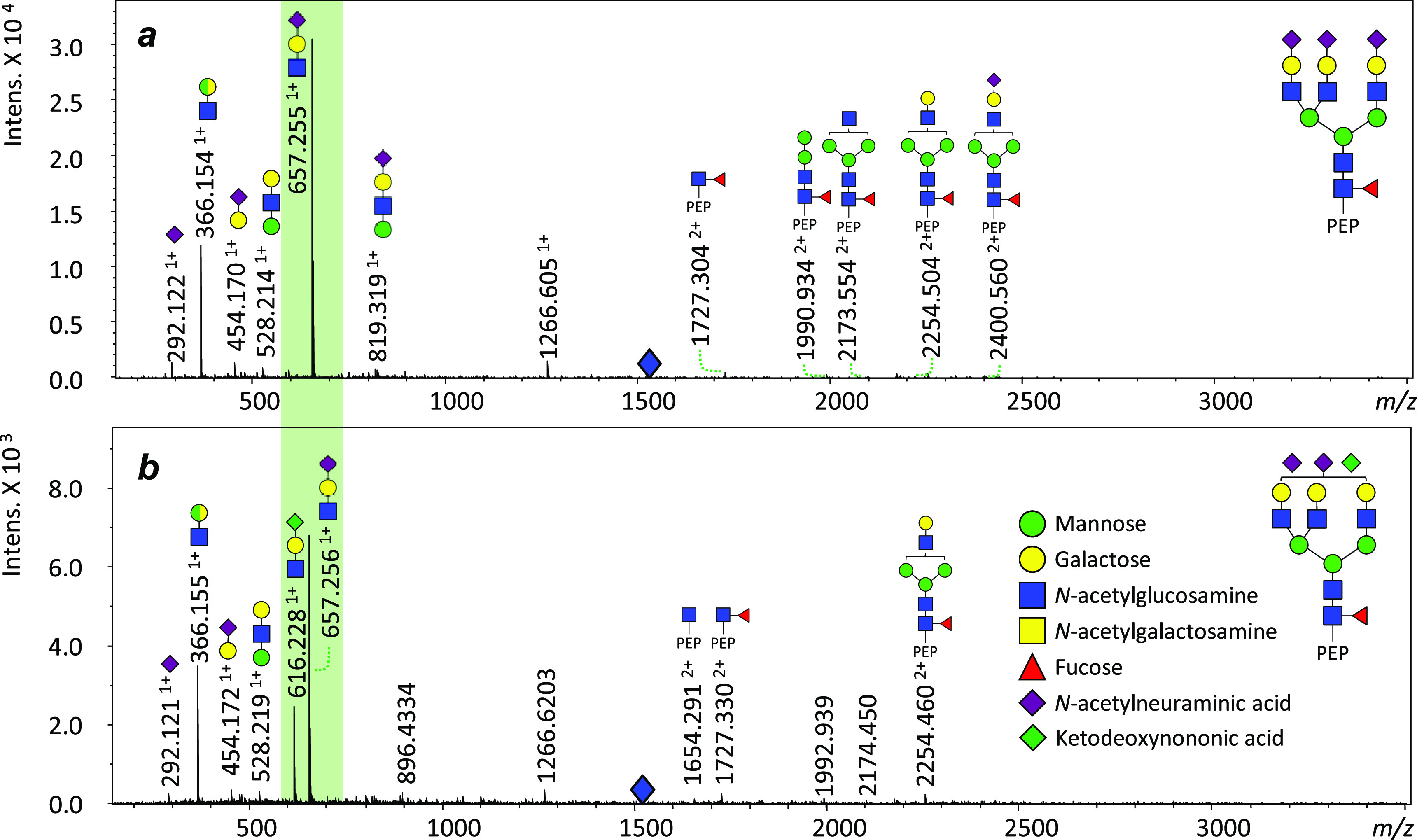
Identification of Kdn-containing *N*-glycans in seminal plasma PAP glycopeptides via tandem MS using CE-MS. (a, b) Tandem MS spectra of glycopeptides H6N5F1S3 and H6N5F1S2K1 on the *N*-glycosylation site N_220_ with peptide backbone VYDPLYCESVHNFTLPSWATEDTMTK. H: hexose. N: *N*-acetylglucosamine. F: fucose. S: *N*-acetylneuraminic acid (Neu5Ac). K: ketodeoxynononic acid (Kdn). Pep: peptide backbone. Green background highlights the significant ions H1N1K1 for Kdn identification. Blue diamonds indicate the parent ions (quadruply charged) of the glycopeptides. The assignment of glycan structures is based on tandem MS spectra.

To test the LOD of the developed assay, different amounts of the PAP standard were spiked, individually, to 1 mL of FUP (0, 20, 50, 100, and 200 ng; Figure S-8). In total, 59, 29, 14, and 0 *N*-glycopeptides with isomer distinction were observed with 200, 100, 50, and 20 ng of spiked PAP, respectively. These results show that the lower LOD of the established assay is between 20 and 50 ng of PAP. To further evaluate the robustness and repeatability of the assay, an intra- and interday validation was performed (*n* = 4 and *n* = 12, respectively). Briefly, 200 ng of the PAP standard was spiked to 1 mL of FUP, followed by an immunoaffinity capture of PAP, tryptic digestion, and measurement by CE-MS. The relative abundance values of PAP glycopeptides were comparable for all three *N*-glycosylation sites (Figures 3 and S-9). For site N_94_, the average RSDs of the top 10 analytes were 5%, 12%, and 14% on days 1, 2, and 3, respectively. For site N_220_, the average RSDs of the top 10 analytes were 8%, 6%, and 25% on days 1, 2, and 3, respectively. Finally, for site N_333_, the average RSDs were 10%, 7%, and 3% on days 1, 2, and 3, respectively.

To demonstrate the applicability of the established assay and to investigate the *N*-glycome of PAP derived from urine, the assay was applied on a pooled DRE urine sample (Figures 2 and S-1 and Table S-3). The glycosylation features of urinary PAP were described above. In addition, a Byonic search (v4.6.1, Protein Metrics, Inc.) of the captured DRE urinary PAP by LC-MS/MS (Orbitrap) showed that PAP was the main protein captured, confirming the success of the urinary PAP assay. Other observed proteins were co-captured from the urine, including hemoglobin subunit OS, semenogelin-1, albumin, semenogelin-2, etc. On the co-captured proteins, no glycans were observed based on the Byonic search (data not shown).

### PAP Glycosylation

A PAP standard derived from seminal plasma was used for the assay development. PAP glycoprofiles of all three sites were characterized by MS and compared to literature reports (Table S-3 and Figure S-10). Consistent with White et al.,^17^ we found that the *N*-glycosylation sites N_94_ and N_220_ were occupied with complex glycans, while site N_333_ contained oligomannosidic glycans. A total of 17 and 13 unique *N*-glycan compositions were identified in seminal PAP for the *N*-glycosylation sites N_94_ and N_220_, respectively. These were all complex-type di-, tri-, and tetraantennary *N*-glycans with and without core fucosylation and with high levels of sialylation. As for site N_333_, four oligomannosidic *N*-glycans were found, ranging from Man 5 to Man 8. Interestingly, ketodeoxynononic acid (Kdn) on human PAP was observed, similar to our first discovery of Kdn-containing *N*-glycans on PSA (Figures 4 and S-11).^35^ One Kdn-containing glycan was identified on site N_220_ and assigned to the glycan composition H6N5F1S2K1 on the basis of MS/MS data (observed *m*/*z* value was 1518.356^4+^, and the theoretical *m*/*z* value was 1518.364^4+^). Three isomers were observed at relative abundances of 0.8%, 1.2%, and 0.8%. Having found Kdn on PSA^35^ and now on PAP, we expect that more human glycoproteins may carry this modification.

In this study, we characterized the glycosylation of DRE urinary PAP for the first time using the established uPGA (Figures 2, S-1, and S-7). Similar to seminal plasma PAP, the urinary PAP glycoprofile has high micro- and macroheterogeneity. Three *N*-glycosylation sites were detected with complex *N*-glycans attached on the *N*-glycosylation sites N_94_ and N_220_, while site N_333_ carried oligomannosidic *N*-glycans. A total of 28 and 12 unique *N*-glycan compositions were identified in urinary PAP for the *N*-glycosylation sites N_94_ and N_220_, respectively, comprising only complex di-, tri- or tetraantennary *N*-glycans with and without core fucosylation and with overall high levels of sialylation. On the basis of the analyte area, 99% of the glycans on site N_94_ were sialylated, of which 34% were monosialylated, 52% were disialylated, and 13% were trisialylated. The fucosylation level on site N_94_ was 38%. An overall high amount of branching was observed on site N_94_ with 60% diantennary, 29% triantennary, and 8% tetraantennary *N*-glycans. Site N_220_ showed glycosylation features similar to site N_94_ with high levels of sialylation, fucosylation, and high branching. Its fucosylation level was more than two times higher than that of site N_94_. All of the *N*-glycans on site N_220_ were found to be sialylated with 20% being tetrasialylated. Both fucosylation (89%) and branching (36% diantennary, 36% triantennary, and 28% tetraantennary *N*-glycans) were found to be higher than for site N_94_. As for site N_333_, four unique *N*-glycan compositions were found with only oligomannosidic *N*-glycans being observed, varying from Man 5 to Man 8. This is inconsistent with a previous study, where the *N*-glycosylation site N_333_ was shown to be occupied not only with oligomannosidic *N*-glycans but also with complex-type *N*-glycans.^18^ This discrepancy could be related to PCa, as the study by Nyalwidhe et al. investigated PAP from DRE urine pools of aggressive PCa. In our study, more information in regard to sialic acid linkages was acquired by CE-MS via isomer separation.^36^ Sialic acid linkage-specific isomers were separated in the CE capillary based on their size and charge, in which α2,6-sialylated glycans/glycopeptides migrated earlier than their α2,3-sialylated variant. This migration behavior might be related to the slight difference in their p*K*_a_ values (a difference of 3.4 × 10^–2^).^37^ This resulted in the identification of 63, 27, and 4 unique *N*-glycan structures on sites N_94_, N_220_, and N_333_, respectively (Table S-4 and Figure S-12). Our analyses provide considerably more depth and coverage than the reports by White et al. and Nyalwidhe et al. Namely, the latter study analyzed a PAP tryptic digest and observed only three, two, and five *N*-glycan structures for sites N_94_, N_220_, and N_333_, respectively. White et al. also analyzed a PAP tryptic digest and observed only three and two *N*-glycan structures for sites N_94_ and N_333_, respectively; no information was acquired for site N_220_. Their results of chymotrypsin digestion showed three, two, and two glycoforms on the sites N_94_, N_220_, and N_333_, respectively.^17^ Next to glycopeptide analysis, the glycosylation of PAP was further examined by enzymatically releasing the *N*-glycans on three different seminal plasma pools (PCa, BPH, and normal seminal). They reported 21 *N*-glycan structures, and similar to our results, this included two oligomannosidic *N*-glycans and 19 complex-type of *N*-glycans. The identified complex glycans were di-, tri-, and tetraantennary *N*-glycans, which were highly sialylated (mono-, di-, tri-, and tetrasialylation) and core fucosylated.^17^ Additionally, bisecting structures were observed for the *N*-glycosylation sites N_94_ and N_220_ by Nyalwidhe et al. (glycopeptide level).^18^ Tandem MS data showed glycan H6N6F1S3 attaching to the peptide backbone FLNESYK. However, the significant ions of the bisecting structure (H1N3 as well as H1N3F1 being attached to the peptide backbone) were not present. The same glycan was also detected in our study; however, our tandem MS data could not confirm whether this glycan was a hybrid- or complex-type *N*-glycan. Interestingly, in our study the Kdn-containing glycan (H6N5F1S2K1) identified on site N_220_ in seminal plasma PAP was also detected in urinary PAP. Similar to seminal PAP, three isomers were observed, all at low relative abundances (0.9%, 0.9%, and 0.6%; see Figure S-11).

Relatively minor glycosylation differences were observed between seminal plasma PAP (the commercial PAP standard) and PAP derived from DRE urine (Figure S-1). The most abundant *N*-glycans on site N_94_ of PAP were H5N4S2 for DRE urinary PAP, followed by H5N4S1 and H5N4F1S2, and the monosialylated non-fucosylated variant was the most abundant (H5N4S1) for seminal plasma, followed by H5N4S2 and H5N4F1S1. In general, it seems that monosialylated structures were more abundant in the seminal pool, while there was higher sialylation in the urinary pool, such as trisialylation (Figures S-1 and S-7). Whether the observed differences are due to the different biofluid types (seminal plasma versus urine) or the health conditions of the urine sample donors remains unresolved. Paired analysis of biofluids from the same individuals would allow us to address this.

During the study, minor questions were prompted and deserve consideration in upcoming investigations. For example, the general signal intensity of PAP dropped 2–3 fold after 2 weeks of storage at −20 °C (data not shown), particularly affecting the coverage of site N_220_, of which the glycopeptide signals already tended to be low after fresh sample preparation. It is unclear whether the signal drop was due to the instability of the PAP glycopeptides and degradation during storage or rather due to aspecific binding and adsorption effects. As a consequence, PAP digests should be measured immediately or stored at −80 °C for a short time after processing.

Apart from the altered glycosylation, PAP is regaining attention, as cPAP also plays an important role in prostate carcinogenesis and progression^11^ because it can inhibit the growth of androgen-independent prostate cells. This has been demonstrated by increased prostate epithelial cell proliferation and subsequently developed invasive adenocarcinomas in PAP knockout mice.^38^ A study using xenograft animal models further indicated the potential of cPAP as a tumor suppressor in PCa.^11^ Gunia et al. also showed the significant inverse correlation between the expression of cytoplasmic PAP and the histopathologic staging of incidental PCa.^39^ Furthermore, PAP has been successfully applied as a therapeutic target for the treatment of patients with castration-resistant prostate cancer (CRPCa).^40,41^ The autologous cellular immunotherapy named Sipuleucel-T, which uses PAP as a target, was approved by the U.S. Food and Drug Administration (FDA) for the treatment of patients with CRPCa.^40,41^ As described previously, cPAP and sPAP differ in glycosylation,^11^ and there is a lack of studies regarding the characterization of the glycosylation of cPAP. Therefore, it is of interest to investigate the glycosylation features of cPAP and explore their biological roles.

## Conclusions

Several studies have implied the diagnostic biomarker potential of an altered PAP glycosylation in relation to PCa. However, these studies lacked the ability to perform an in-depth characterization to identify low abundant species. In this study, we established uPGA, which allowed us to distinguish α2,3- from α2,6-linked sialylation, and its potential was demonstrated on pooled samples, revealing a LOD between 20 and 50 ng/mL and the high glycosylation complexities of sites N_94_ and N_220_. Overall, this study provides an important stepping stone to further verify the previous discoveries and evaluate the clinical and diagnostic potential of PAP glycosylation features on an individual level in large sample cohorts.

## Supporting Information Available

Tables S-1–S-6: PAP information on the pool of DRE urine samples, precursor ions exclusion list applied during tandem MS analysis of DRE urinary PAP, identification list of seminal plasma PAP and DRE urinary PAP glycans with CE-MS/LC-MS, relative abundance of tryptic PAP glycopeptides per glycosylation site, peptide backbones of tryptic PAP glycopepides, and optimization of PAP digestion with different types of trypsin at different enzmye:protein ratios (XLSX)
Materials and methods, relative abundance of observed glycopeptides per glycosylation sites on PAP, anti-PAP antibodies coupling to HCS beads, reduced SDS-PAGE gels of PAP capture with varying concentrations of FA as the elution buffer and different amounts of HCS beads, comparison of the glycoprofiles of PAP captured by anti-PAP I and anti-PAP II and non-captured PAP standard, comparison of proteolytic digestion of PAP using three types of trypsin, glycosylation signatures of DRE urinary PAP and seminal plasma PAP on sites N_94_, N_220_, and N_333_, LOD experiment of the developed uPGA method, intra- and interday validation of the developed uPGA, identification of seminal plasma PAP and DRE urinary PAP glycopeptides via tandem MS using CE-MS, and relative abundance of observed Kdn-containing glycopeptides (PDF)

## Abbreviations

DRE: Digital rectal examination
CAA: Chloroacetamide
CE: Capillary electrophoresis
cPAP: cellular prostatic acid phosphatase
CRPCa: Castration-resistant prostate cancer
DTT: DL-dithiothreitol
FA: Formic acid
FUP: Female urine pool
IAA: Iodoacetamide
HCS: High-capacity streptavidin agarose resins
LC: Liquid chromatography
LE: Leading electrolyte
LOD: Limit of detection
MALDI: Matrix-assisted laser desorption/ionization
MS: Mass spectrometry
N: Asparagine (Asn)
ON: Overnight
pI: Isoelectric point
PSA: Prostate-specific antigen
PAP: Prostatic acid phosphatase
PBS: Phosphate-buffered saline
PCa: Prostate cancer
rt: Room temperature
RSD: Relative standard deviation
SGM: Sequence grade modified
sPAP: Secreted prostatic acid phosphatase
TOF-MS: Time-of-flight mass spectrometry
uPGA: Urinary PAP glycoproteomic assay
Q: Glutamine (Gln)
