# Supplementary material for: In-Depth Glycoproteomic Assay of Urinary Prostatic Acid Phosphatase
Source: ACS Meas Sci Au. 2023 Dec 8;4(1):117–26. doi: 10.1021/acsmeasuresciau.3c00055 (PMC10885330; doi:10.1021/acsmeasuresciau.3c00055)
Supplement: Supplementary file 2 — tg3c00055_si_002.pdf [file tg3c00055_si_002.pdf]

SUPPORTING INFORMATION

In-depth Glycoproteomic Assay of Urinary Prostatic Acid Phosphatase

Wei Wang[1], Carmen R. de Nier[1], Manfred Wuhrer[1], Guinevere S.M. Lageveen-Kammeijer[1,2*]

[1] Leiden University Medical Center, Center for Proteomics and Metabolomics, Leiden, 2300 RC, the Netherlands

[2] University of Groningen, Groningen Research Institute of Pharmacy, Groningen, 9713 AV, The Netherlands

***Correspondence:** Guinevere S.M. Lageveen-Kammeijer, Faculty of Science and Engineering, Analytical Biochemistry — Groningen Research Institute of Pharmacy, Antonius Deusinglaan 1, 9713 AV Groningen, The Netherlands; g.s.m.kammeijer@rug.nl; Tel: +316 297 999 66

Table of Contents

S-1. SUPPORTING INFORMATION – MATERIALS AND METHODS 3

S-1.1 Chemicals 3

S-1.2 SDS-PAGE 3

S-1.3 RP-nanoLC-MS (Q-TOF) 4

S-1.4 RP-nanoLC-MS/MS (Orbitrap) 4

S-2. SUPPORTING INFORMATION – FIGURES 5

Figure S-1. Relative abundance of observed glycopeptides per glycosylation sites on PAP. 5

Figure S-2. Anti-PAP antibodies (I and II) coupling to high-capacity streptavidin agarose resins (HCS beads). 6

Figure S-3. Reduced SDS-PAGE gels of PAP capture to test the capturing efficiency (binding of PAP to anti-PAP beads) with FA of different concentrations as elution buffer. 7

Figure S-4. Reduced SDS-PAGE gels of PAP capture to test the capturing efficiency (binding of PAP to anti-PAP beads) with different amount of HCS beads. 8

Figure S-5. Capture bias examined by comparing the glycoprofiles of PAP captured by anti-PAP I, captured by anti-PAP II and non-captured PAP standard (POS). 9

Figure S-6.1 Comparison of proteolytic digestion of PAP using three types of trypsin. Several missed cleavages were observed for 10

Figure S-6.2 Comparison of proteolytic digestion of PAP using three types of trypsin. 11

Figure S-7. Glycosylation signatures of DRE urinary PAP and seminal plasma PAP on glycosylation sites N_{94}, N_{220} and N_{333}. 12

Figure S-8. Limit of detection (LOD) experiment of the developed uPGA method. 13

Figure S-9. Intra-and interday validation of the developed uPGA showing relative abundance of observed glycopeptides of the three glycosylation sites. 14

Figure S-10. The identification of seminal plasma PAP glycopeptides via tandem MS using CE-MS. 15

Figure S-11. Relative abundance of observed Kdn-containing glycopeptide H6N5F1S2K1-VYDPLYCESVHNFTLPSWATEDTMTK glycopeptides on glycosylation site N_{220} of PAP derived from DRE urine and seminal plasma. 23

Figure S-12. The identification of DRE urinary PAP glycopeptides via tandem MS using CE-MS. 24

S-1. SUPPORTING INFORMATION – MATERIALS AND METHODS

S-1.1 Chemicals

Milli-Q water (MQ) was used for all buffer preparations and washing steps and was generated from a Purelab Chorus ELGA system (Millipore, Amsterdam, the Netherlands). Ammonium bicarbonate (ABC), iodoacetamide (IAA), DL-dithiothreitol (DTT), Tris(2-carboxyethyl)phosphine hydrochloride solution (TCEP), Chloroacetamide (CAA) and TPCK treated trypsin were purchased from Sigma-Aldrich (Steinheim, Germany). Sequence grade modified trypsin (SGM), trypsin gold and trypsin platinum were obtained from Promega (Madison, WI). Sodium azide (NaN_3) was acquired from pharmacy AZL (Leiden, the Netherlands) and beta-mercaptoethanol (βME) from VWR Life (Pennsylvania, USA). Acetonitrile gradient grade for LC-MS (MeCN), methanol (MeOH), sodium phosphate dibasic dihydrate ($Na_2HPO_4{\cdot}2H_2O$), monopotassium phosphate (KH_2PO_4), sodium chloride (NaCl) and acetic acid (HAc, purity 99.8%-100.5%) were obtained from Merck (Darmstadt, Germany). One time concentrated (1x) PBS was prepared by diluting 5x PBS with MQ. The 5x PBS consisted out of 0.16 M Na_2HPO_4, 0.02 M KH_2PO_4 and 0.73 M NaCl, with a pH of 7.2, formic acid (FA) and LC-MS grade water were purchased from Fluka (Steinheim, Germany). Precision Plus Protein All Blue Standards was obtained from Bio-Rad (Lunteren, The Netherlands). Der Blaue Jonas was acquired from German Research Products (Amper, Germany). NuPAGE LDS sample buffer (4x), 20x diluted NuPAGE MOPS (3-morpholinopropanesulfonic acid) running buffer, Colloidal Blue Staining Kit, NuPAGE (4 to 12% Bis-Tris, 1.0 mm, Mini Protein gel 15-well and 4 to 12%, Bis-Tris, 1.5 mm, Mini Protein gel 10-well) were purchased from Thermo Fisher Scientific (Waltham, MA). RapiGest SF Surfactant was purchased from Waters Corporation (Milford, MA). Prostatic acid phosphatase (PAP) standard derived from human seminal fluid was acquired from Lee BioSolutions (St. Louis, MO). High-capacity streptavidin agarose resins (HCS beads) from Thermo Scientific (Rockford, IL). Anti-PAP antibodies were kindly provided by Roche Diagnostics (Penzberg, Germany).

S-1.2 SDS-PAGE

For all SDS-PAGE experiments, samples were dried and reconstituted in 15 µL loading buffer which contains 4x diluted NuPAGE LDS sample buffer with 5% βME. For denaturation, samples were heated for 10 min at 70 ^{o}C, followed by a centrifugation at 10,000 rpm for 1 min. Samples were loaded onto an SDS-PAGE gel which was run for approximately 1 h at 180 V using MOPS SDS running buffer (1x). The gels were stained with Colloidal Blue (Thermo Fisher Scientific, Waltham, MA) or with Der Blaue Jonas (German Research Products, Amper, Germany) for 30 min followed by de-staining in MQ for 3-5 washing steps. To enable a relative quantification of the intensity of the PAP protein bands on the SDS-PAGE, the software GelAnalyzer (2010a freeware) was used.

S-1.3 RP-nanoLC-MS (Q-TOF)

Measurements were performed on an Ultimate 3000 RSLCnano system (Thermo Scientific, Breda, the Netherlands) equipped with an Acclaim PepMap 100 C18 HPLC trap column (100 µm x 20 mm, particle size 5 µm, Thermo Scientific) and an Acclaim PepMap 100 C18 LC analytical column (75 µm x 150 mm, particle size 2 µm, Thermo Scientific). The separation was performed with a multi-step gradient consisting out solvent A (H_20/0.1% FA/0.1% CAN; *v/v/v*) and solvent B (95% MeCN/5% H_2O containing 0.1% FA; *v/v/v*). For an optimal separation, various gradients were investigated. The final LC method starts with solvent B at 1% for 5 min, followed by a linear increase to 40% over a 45 min period. An additional increase to 80% (solvent B) followed in 1 min and was kept constant for 4 min. Afterwards the percentage of solvent B was reduced to 1% in 1 min and the column was reconditioned with 1% solvent B for 34 min. The flow rate during the whole procedure was set at 0.7 µL/min. The LC system was coupled to a maXis HD quadrupole time-of-flight-MS (q-TOF-MS, Bruker Daltonics) via an ESI interface, equipped with CaptiveSpray and nanoBooster (Bruker Daltonics), using MeCN as the dopant gas. The MS was operated in stepping-energy CID mode and a full MS scan was recorded in the *m/z* range of 500 to 2500. The glass capillary voltage was set at 1200 V.

S-1.4 RP-nanoLC-MS/MS (Orbitrap)

The tryptic PAP glycopeptides were measured on a nanoLC-MS system composed of an Easy nLC1200 gradient HPLC system (Thermo, Bremen, Germany) coupled to an Orbitrap Fusion LUMOS MS (Thermo). The generated PAP glycopeptides from DRE urine derived PAP followed by SGM tryptic digestion were 10 times diluted in 10 µL of 0.1% FA. With solvent A (water/FA; 100/0.1; *v/v/v*), sample was loaded onto an in-house packed C18 precolumn (100 µm × 15 mm; Reprosil-Pur C18-AQ 3 µm, Dr. Maisch, Ammerbuch, Germany), and separated on a homemade analytical nanoLC column (30 cm × 75 µm; Reprosil-Pur C18-AQ 3 µm). For the elution of the analytes, a linear gradient was set increase from 2% to 40% in 30 min for solvent B (water/MeCN/FA; 20/80/0.1; *v/v/v*). The nanoLC column was drawn to a tip of approximately 5 µm and acted as the electrospray needle of the MS source. The MS1 spectra were acquired with a mass range of *m/z* 350–1600 at the automatic gain control (AGC) standard target value combined with a maximum accumulation time of 50 ms. The resolution setting for the MS1 scans was set at 120,000. A dynamic exclusion was set for 10 s with a single repeat count, and charge states in the range 2–5 were included for the MS/MS. The resolution of the MS/MS scans was 30,000 at an AGC target of 5×10^4 with a maximum fill time of 60 ms. MS/MS spectra were generated from precursors isolated with the quadrupole with an isolation width of 1.2 Da at a scan range (*m/z*) of 190–2000. Upon detection of the *N*-acetylhexosamine (HexNAc) oxonium ion at *m/z* 204.087 (with a tolerance of 10 ppm), a higher energy collisional dissociation (HCD) scan was triggered with stepped normalized energies of 25%, 32%, and 39%, at a normalized AGC target of 200% in combination with a maximum fill time in auto mode. The scan range of triggered tandem MS is *m/z* 120-2400.

S-2. SUPPORTING INFORMATION – FIGURES

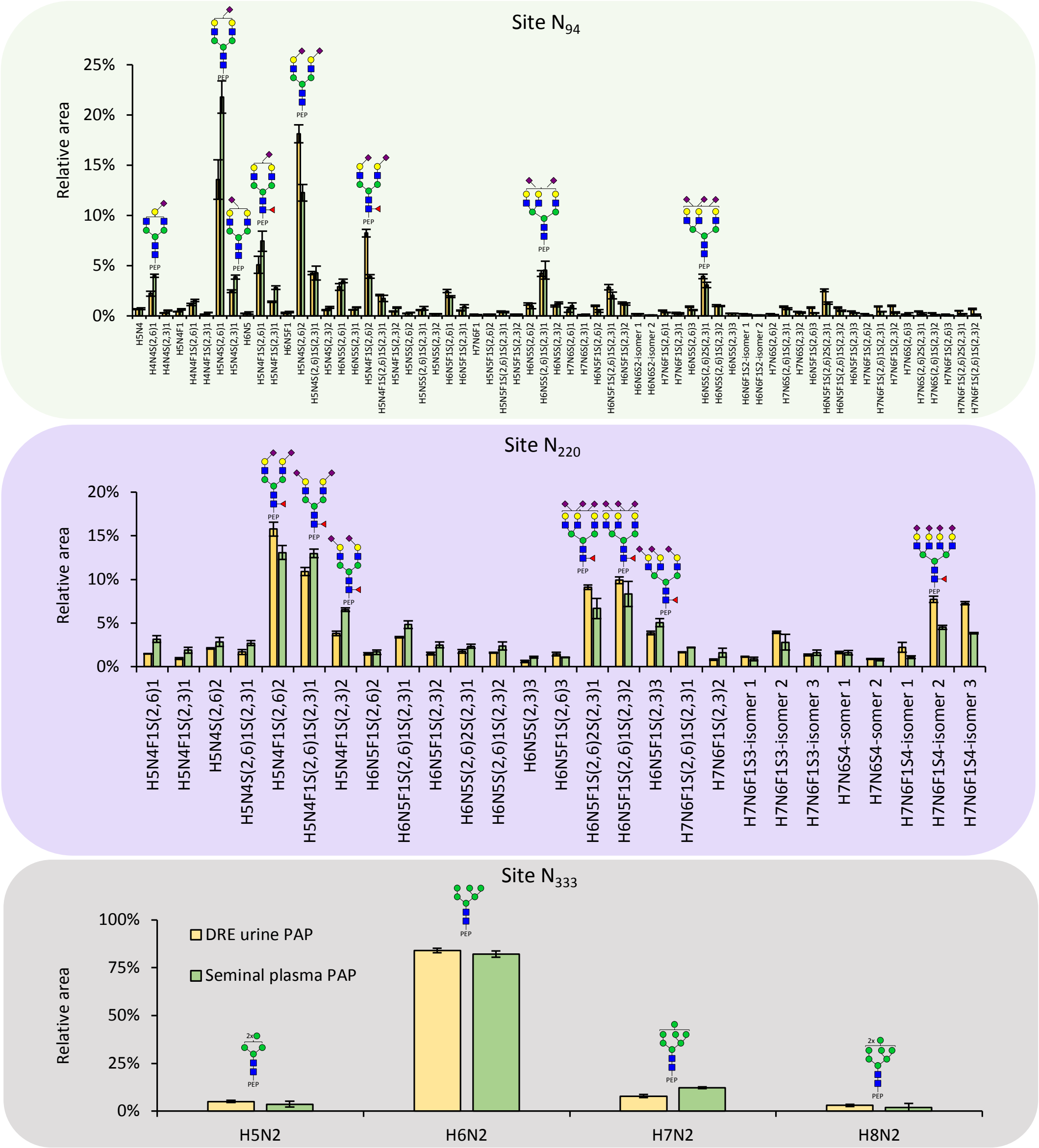

<table>
<tr><td rowspan="2">RSD</td><td colspan="2">DRE urine PAP</td><td colspan="2">Seminal plasma PAP</td></tr>
<tr><td>AVG</td><td>AVG (top 10 analytes)</td><td>AVG</td><td>AVG (top 10 analytes)</td></tr>
<tr><td>Site N$_{94}$</td><td>9.8%</td><td>8.3%</td><td>14.9%</td><td>9.1%</td></tr>
<tr><td>Site N$_{220}$</td><td>7.5%</td><td>4.0%</td><td>13.2%</td><td>8.5%</td></tr>
<tr><td>Site N$_{333}$</td><td>11.7%</td><td>–</td><td>36.4%</td><td>–</td></tr>
</table>

Figure S-1. Relative abundance of observed glycopeptides per glycosylation sites on PAP. *N*-glycan structures are shown for a few of the most abundant *N*-glycans per site. RSD: relative standard deviation. H: hexose. N: *N*-acetylglucosamine. F: fucose. S: *N*-acetylneuraminic acid (Neu5Ac). The assignment of glycan structures is based on tandem MS spectra.

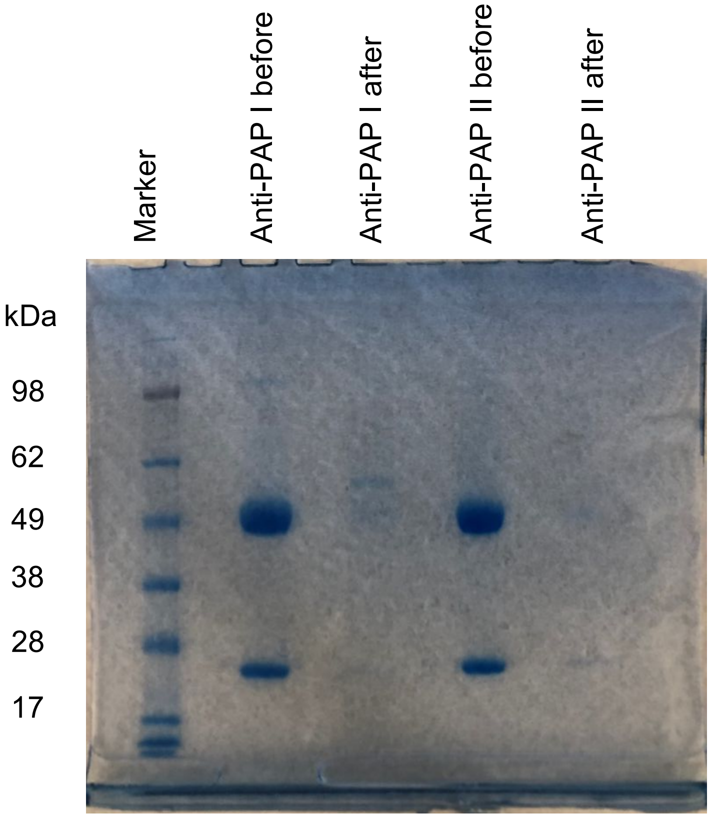

Figure S-2. Anti-PAP antibodies (I and II) coupling to high-capacity streptavidin agarose resins (HCS beads). The coupling efficiency was determined by comparing the same volume prior (approximately 9 µg of antibodies) and after coupling the antibodies by SDS-PAGE analysis.

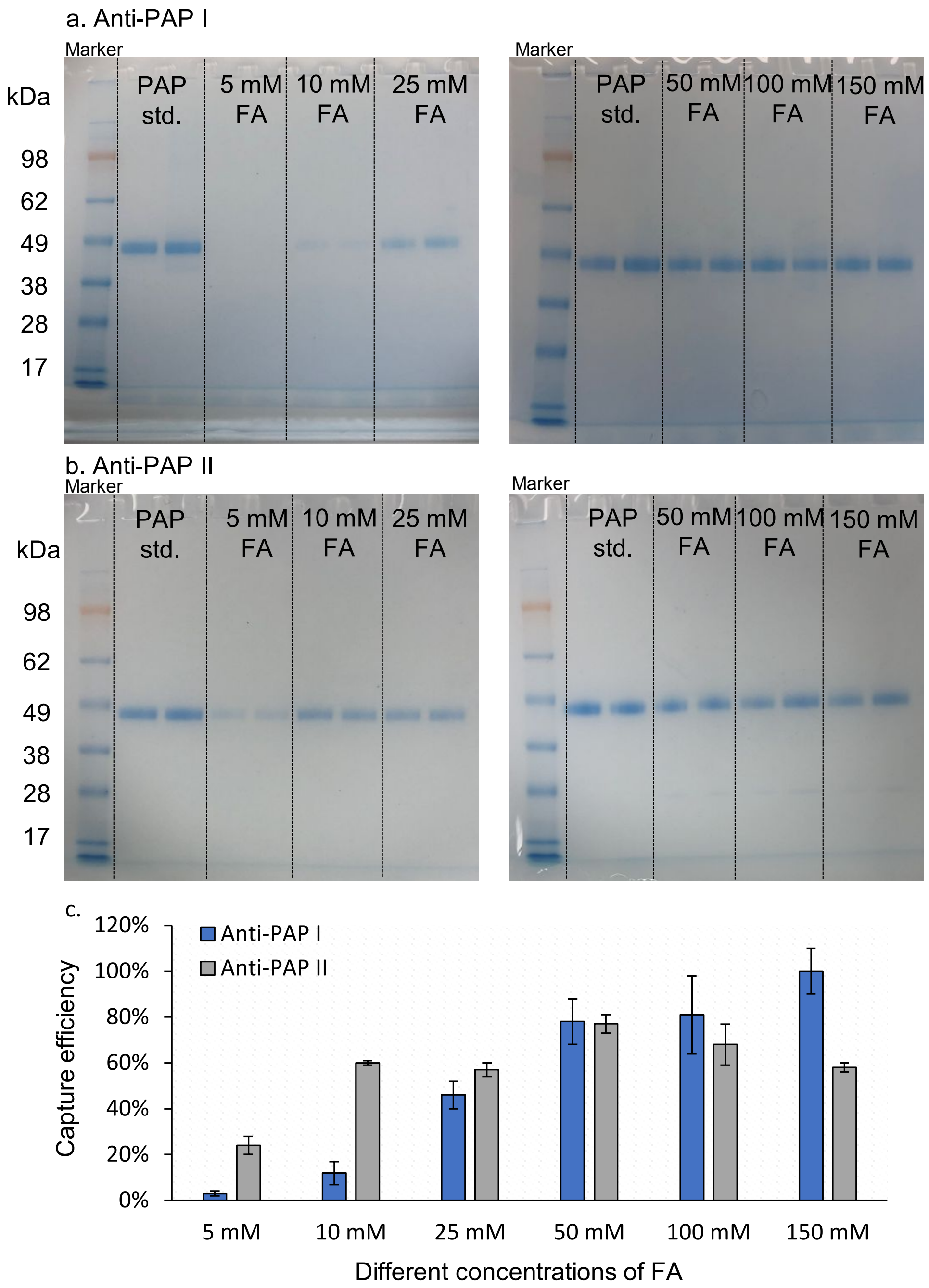

Figure S-3. Reduced SDS-PAGE gels of PAP capture to test the capturing efficiency (binding of PAP to anti-PAP beads) with FA of different concentrations as elution buffer. The PAP standard (1.5 µg) was directly loaded onto the gel as a control. Same amounts of PAP were added to one mL of female urine pool (FUP) and captured by affinity purification using **(a)** anti-PAP antibody I and **(b)** anti-PAP antibody II. A series of FA concentrations (5 mM, 10 mM, 25 mM, 50 mM, 100 mM and 150 mM) for the elution buffer were evaluated for an optimal affinity purification procedure. **(c)** compares the performance (PAP recovery) of the two anti-PAP antibodies. Error bar represents the standard deviation of the duplicates.

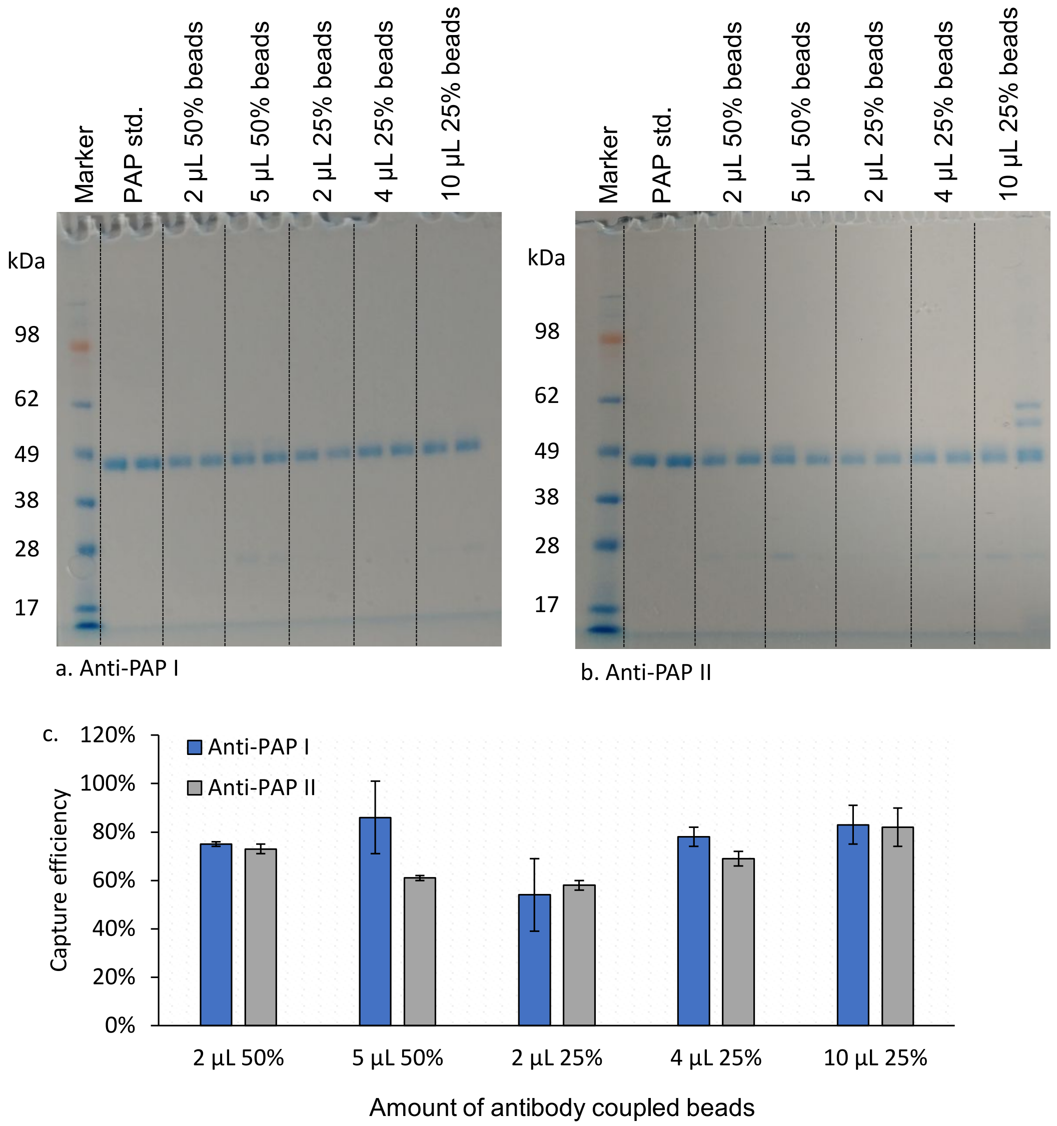

Figure S-4. Reduced SDS-PAGE gels of PAP capture to test the capturing efficiency (binding of PAP to anti-PAP beads) with different amount of HCS beads. The PAP standard (1.5 µg) was directly loaded onto the gel as a control. Same amounts of PAP were added to one mL of FUP and captured by affinity purification using **(a)** anti-PAP antibody I and **(b)** anti-PAP antibody II. **(c)** compares the performance (PAP recovery) of the two anti-PAP antibodies. Error bar represents the standard deviation of the duplicates.

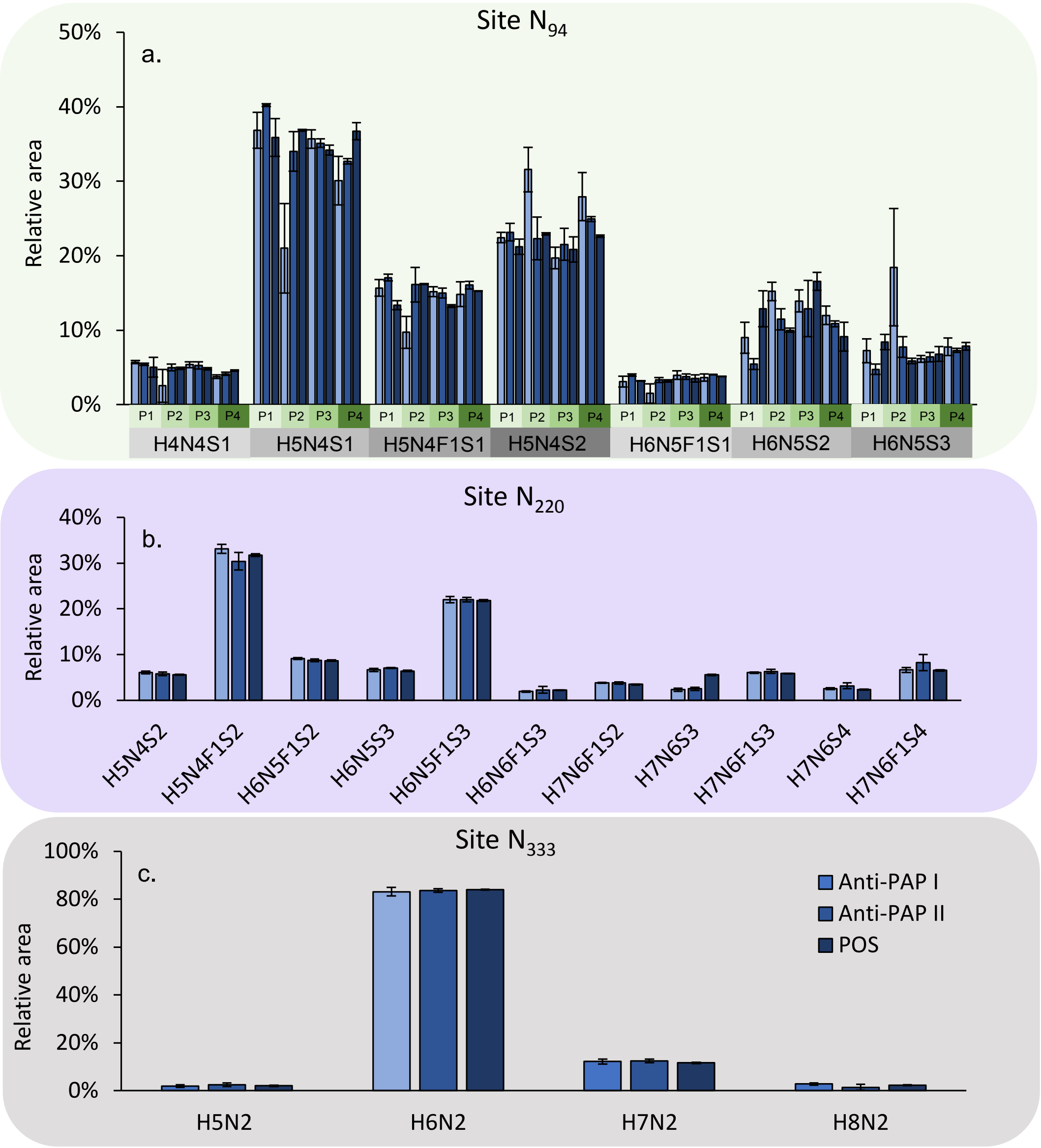

Figure S-5. Capture bias examined by comparing the glycoprofiles of PAP captured by anti-PAP I, captured by anti-PAP II and non-captured PAP standard (POS). (a, b, c) showing the PAP glycoprofiles on sites N_{94}, N_{220} and N_{333},respectively. On site N_{94}, missed cleavages occurred as four different peptide backbones were observed. P1 represents FL$\mathbf{N_{94}}$ESYK. P2 represents KFL$\mathbf{N_{94}}$ESYKHEQVYIR. P3 represents KFL$\mathbf{N_{94}}$ESYK and P4 represents FL$\mathbf{N_{94}}$ESYKHEQVYIR. Error bar represents the standard deviation of the triplicates. H: hexose. N: *N*-acetylglucosamine. F: fucose. S: *N*-acetylneuraminic acid (Neu5Ac).

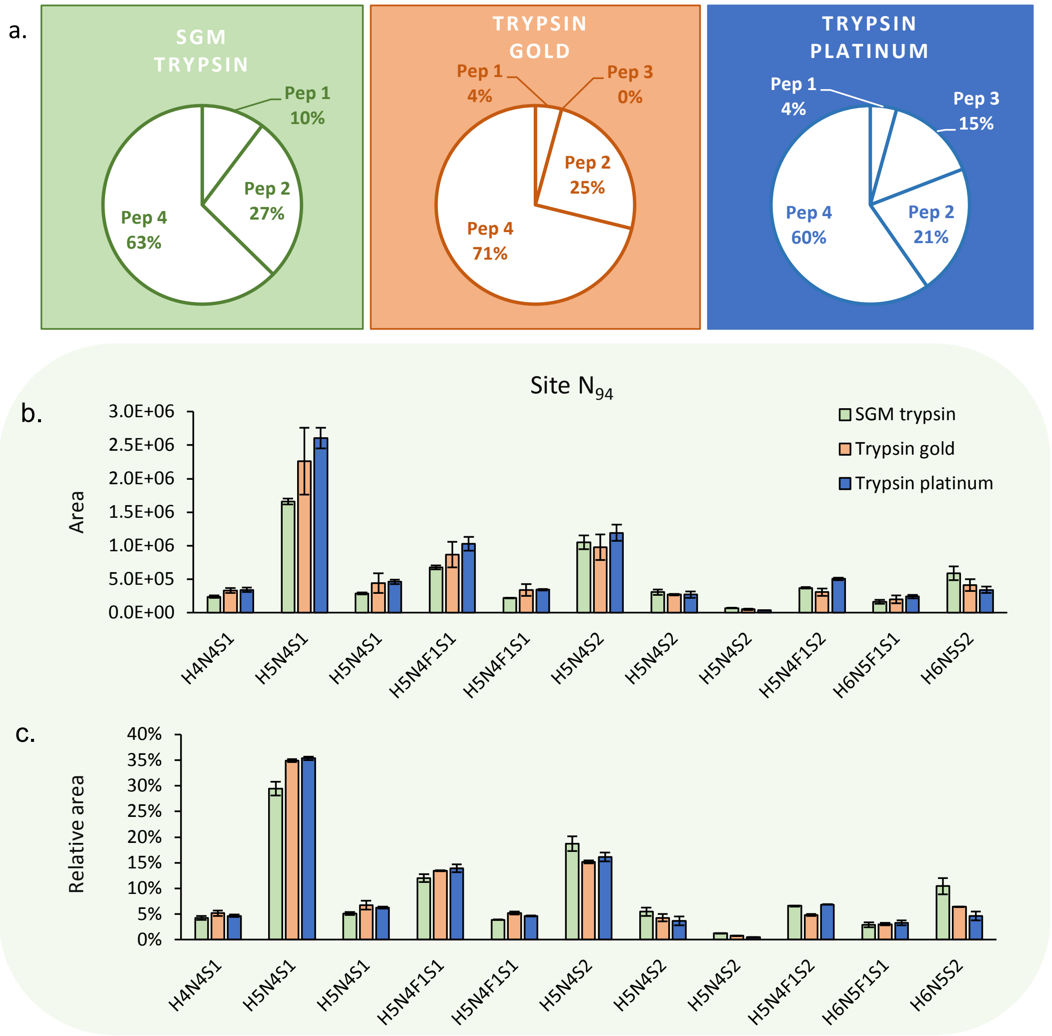

Figure S-6.1 Comparison of proteolytic digestion of PAP using three types of trypsin. Several missed cleavages were observed for glycosylation site N_{94}. **(a)** Shows the ratios of PAP glycopeptides with four different peptide backbones. **(b)** Shows absolute and **(c)** shows relative area of PAP glycopeptides detected on glycosylation site N_{94}. Pep 1:FL$\mathbf{N_{94}}$ESYK. Pep 2: KFL$\mathbf{N_{94}}$ESYKHEQVYIR. Pep 3: KFL$\mathbf{N_{94}}$ESYK. Pep 4: FL$\mathbf{N_{94}}$ESYKHEQVYIR. Trypsin:PAP of 1:5 was used. Error bar represents the standard deviation of the triplicates. H: hexose. N: *N*-acetylglucosamine. F: fucose. S: *N*-acetylneuraminic acid (Neu5Ac). Note: only analytes detected in all samples, despite the type of trypsin used, are shown.

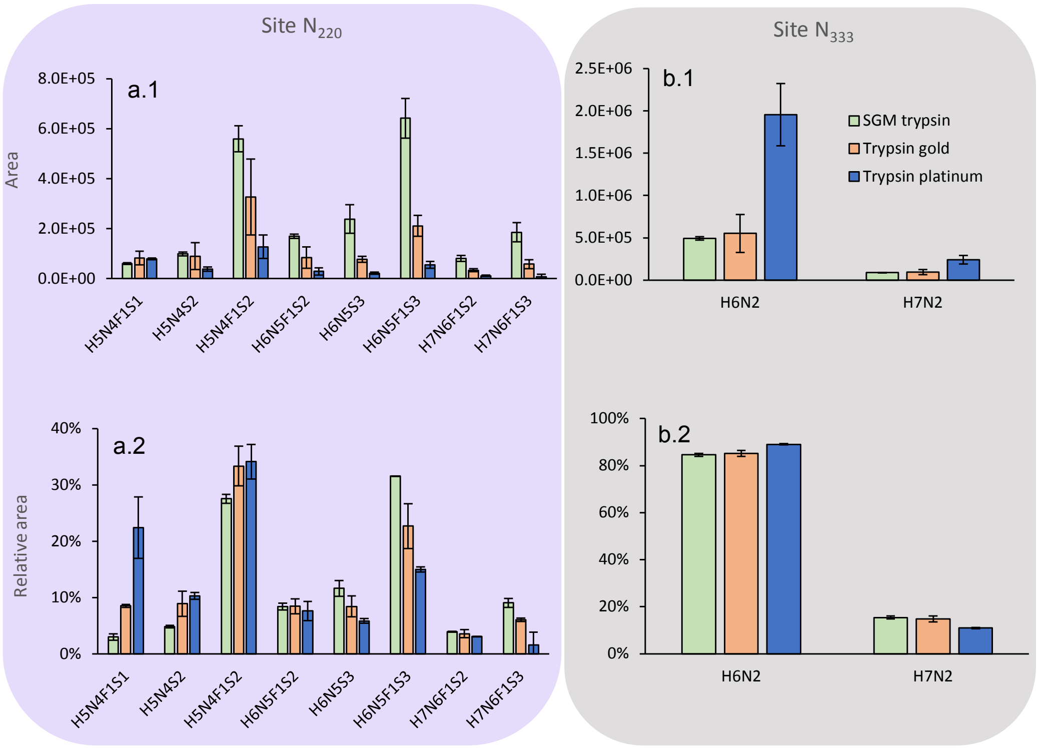

c.

AVG RSD of relative area	Site N_{94}	Site N_{220}	Site N_{333}
SGM trypsin	8.1%	7.0%	2.5%
Trypsin gold	5.8%	14.9%	5.3%
Trypsin platinum	7.6%	26.8%	1.2%

Figure S-6.2 Comparison of proteolytic digestion of PAP using three types of trypsin. (a.1) Shows absolute and **(a.2)** shows relative area of PAP glycopeptides detected on glycosylation site N_{220}. **(b.1)** Shows absolute and **(b.2)** shows relative area of PAP glycopeptides detected on glycosylation site N_{333}. **(c)** Illustrates the average relative standard deviation (AVG RSD) of detected glycopeptides per glycosylation sites with per type of trypsin. Trypsin:PAP of 1:5 was used. Error bar represents the standard deviation of the triplicates. H: hexose. N: *N*-acetylglucosamine. F: fucose. S: *N*-acetylneuraminic acid (Neu5Ac). Note: only analytes detected in all samples, despite the type of trypsin used, are shown.

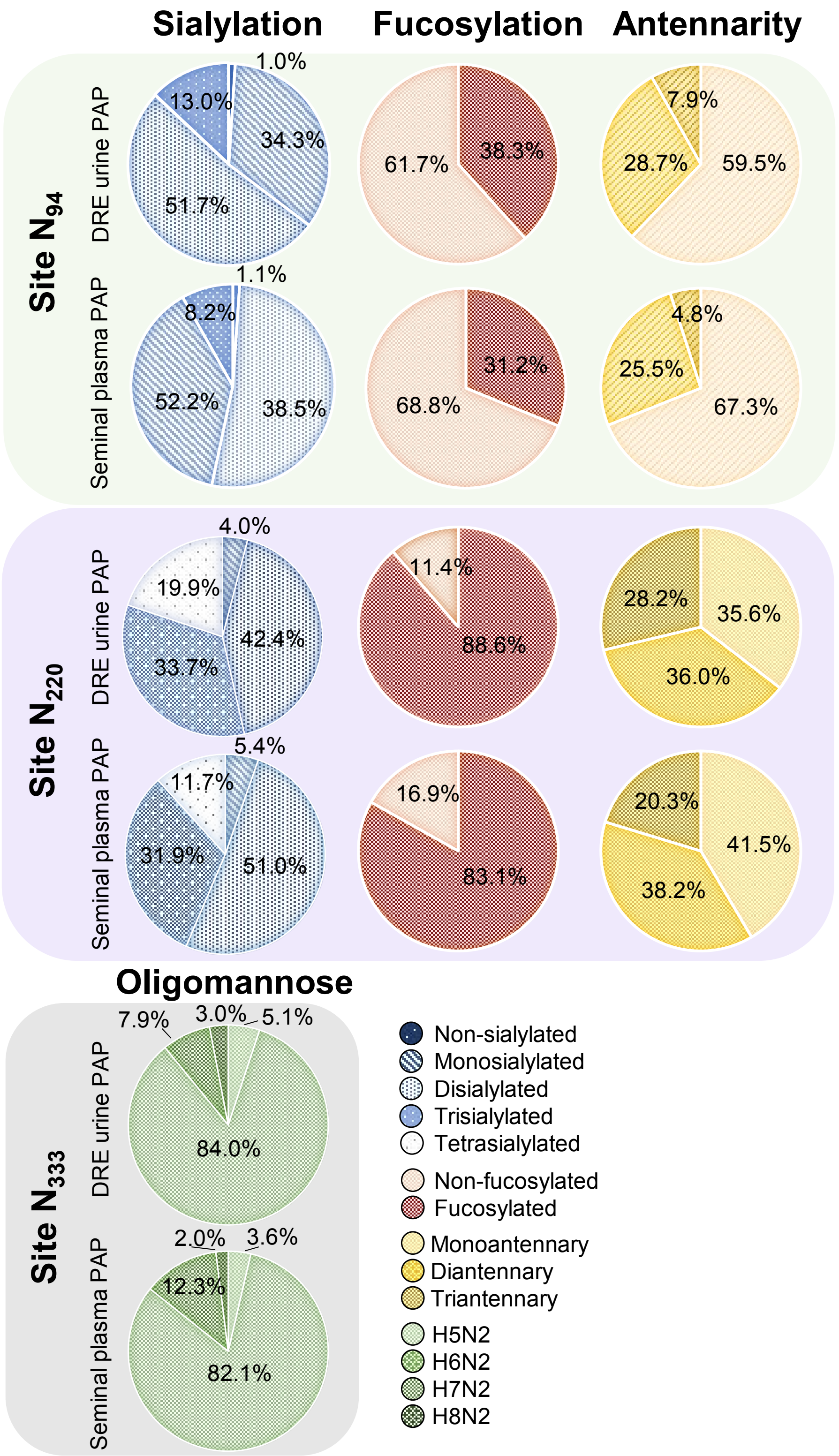

Figure S-7. Glycosylation signatures of DRE urinary PAP and seminal plasma PAP on glycosylation sites N_{94}, N_{220} and N_{333}.

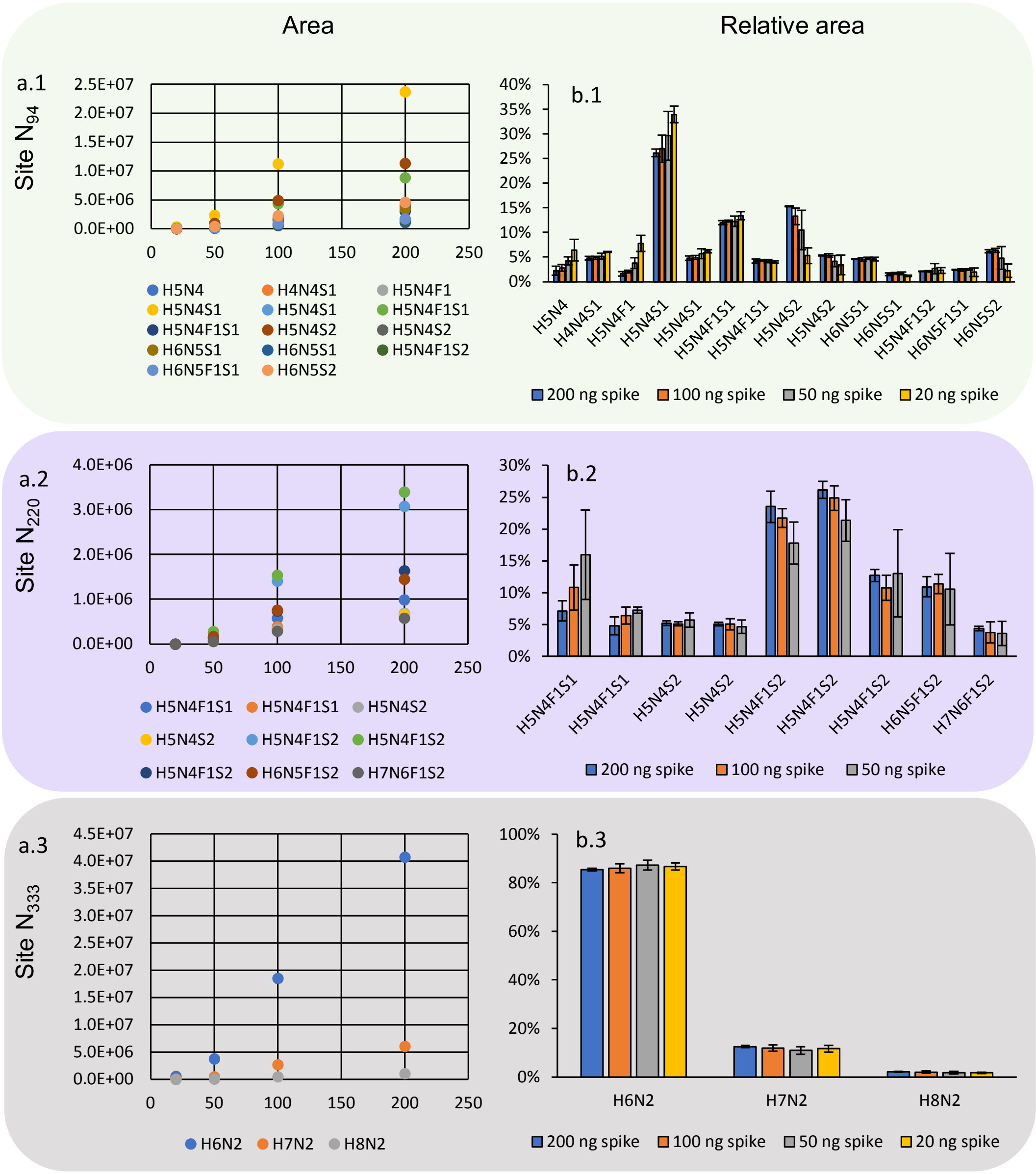

Figure S-8. Limit of detection (LOD) experiment of the developed uPGA method. A series of different amount of PAP standards (20 ng, 50 ng, 100 ng and 200 ng) were spiked to one mL of FUP. The spiked PAP was captured and digested with SGM trypsin, followed by CE-MS measurement. **(a.1, a.2 and a.3)** Presents the analytes areas comparing different amount (ng) of spiked PAP on sites N_{94}, N_{220} and N_{333} respectively. **(b.1, b.2 and b.3)** Present the relative area of detected glycopeptides comparing different amount (ng) of spiked PAP on sites N_{94}, N_{220} and N_{333} respectively. H: hexose. N: *N*-acetylglucosamine. F: fucose. S: *N*-acetylneuraminic acid (Neu5Ac).

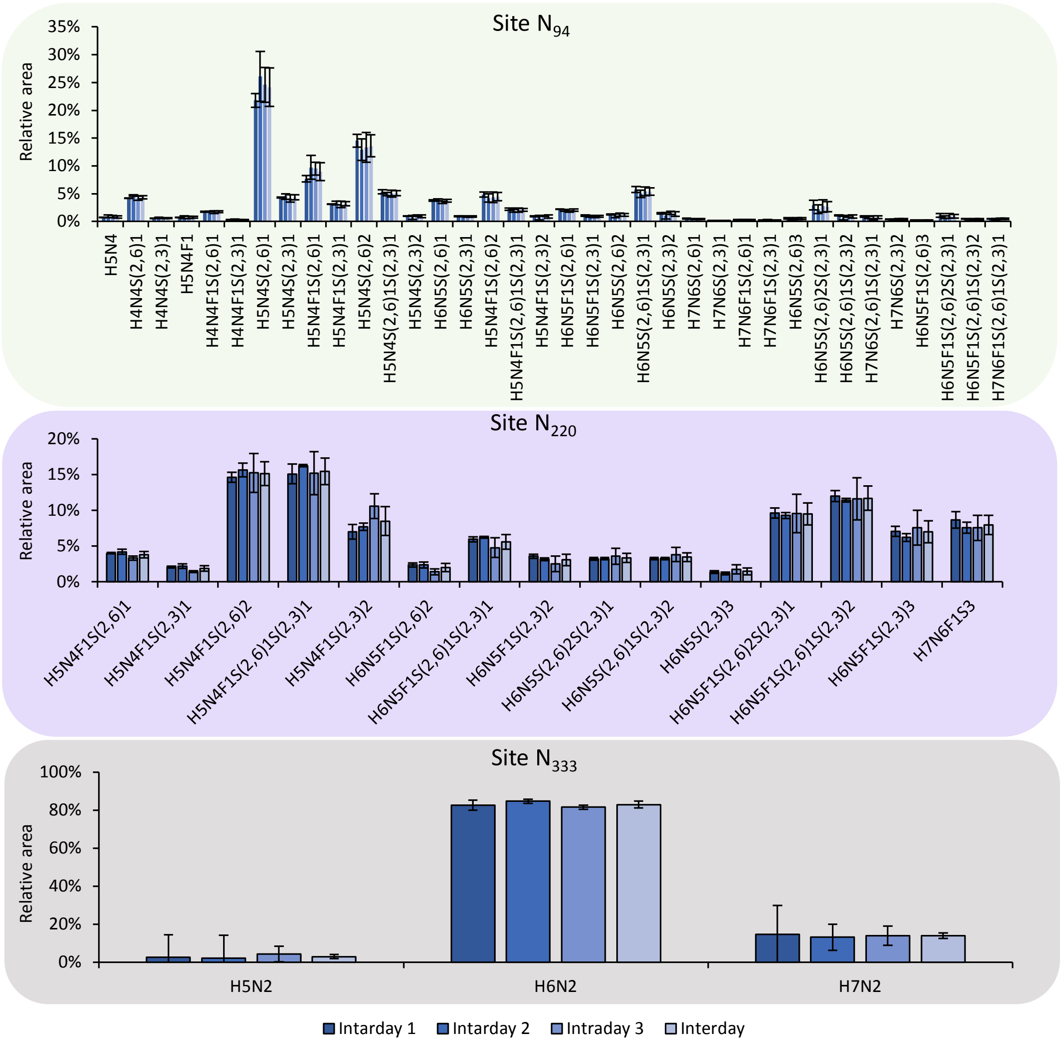

RSD	Intraday 1		Intraday 2		Intraday 3		Interday	
	AVG	AVG (top 10 analytes)	AVG	AVG (top 10 analytes)	AVG	AVG (top 10 analytes)	AVG	AVG (top 10 analytes)
Site N_{94}	9.5%	5.3%	25.8%	12.1%	18.9%	14.2%	20.3%	12.1%
Site N_{220}	8.2%	8.1%	7.7%	5.5%	25.2%	25.0%	19.4%	16.5%
Site N_{333}	9.9%	–	6.7%	–	3.4%	–	15.5%	–

Figure S-9. Intra-and interday validation of the developed uPGA showing relative abundance of observed glycopeptides of the three glycosylation sites. Seminal plasma PAP standard was spiked into FUP, an in-solution digestion was performed and was used to test the repeatability (intraday, N = 4) and intermediate precision (interday, N = 12) of the uPGA by CE-MS. H: hexose. N: *N*-acetylglucosamine. F: fucose. S: *N*-acetylneuraminic acid (Neu5Ac).

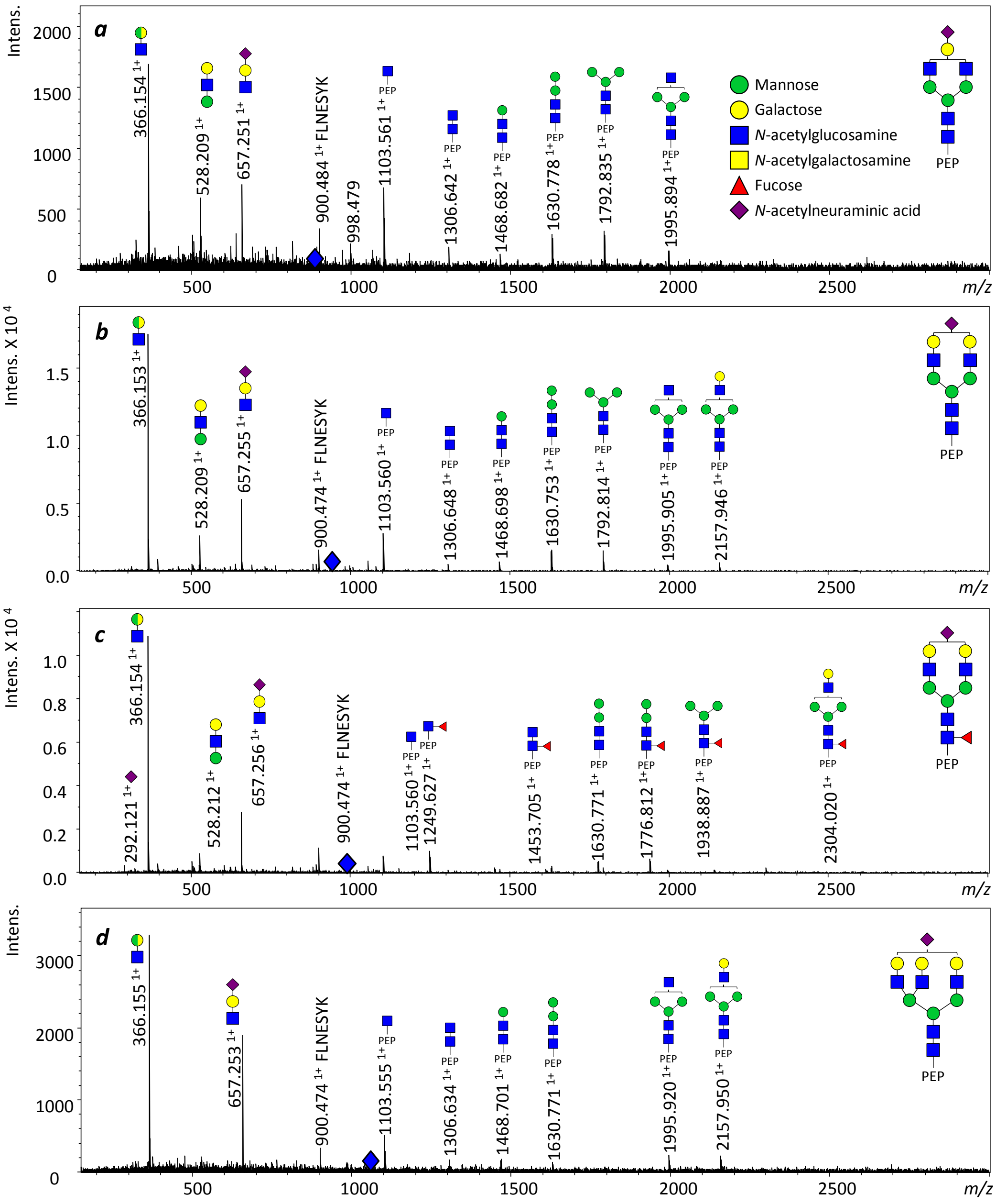

Figure S-10. The identification of seminal plasma PAP glycopeptides via tandem MS using CE-MS. (a-d) Tandem MS spectra of glycopeptides H4N4S1, H5N4S1, H5N4F1S1 and H6N5S1 on glycosylation site N_{94} with peptide backbone FLNESYK. Blue diamond marks the precursor ion. H: hexose. N: *N*-acetylglucosamine. F: fucose. S: *N*-acetylneuraminic acid (Neu5Ac). The assignment of glycan structures is based on tandem MS spectra.

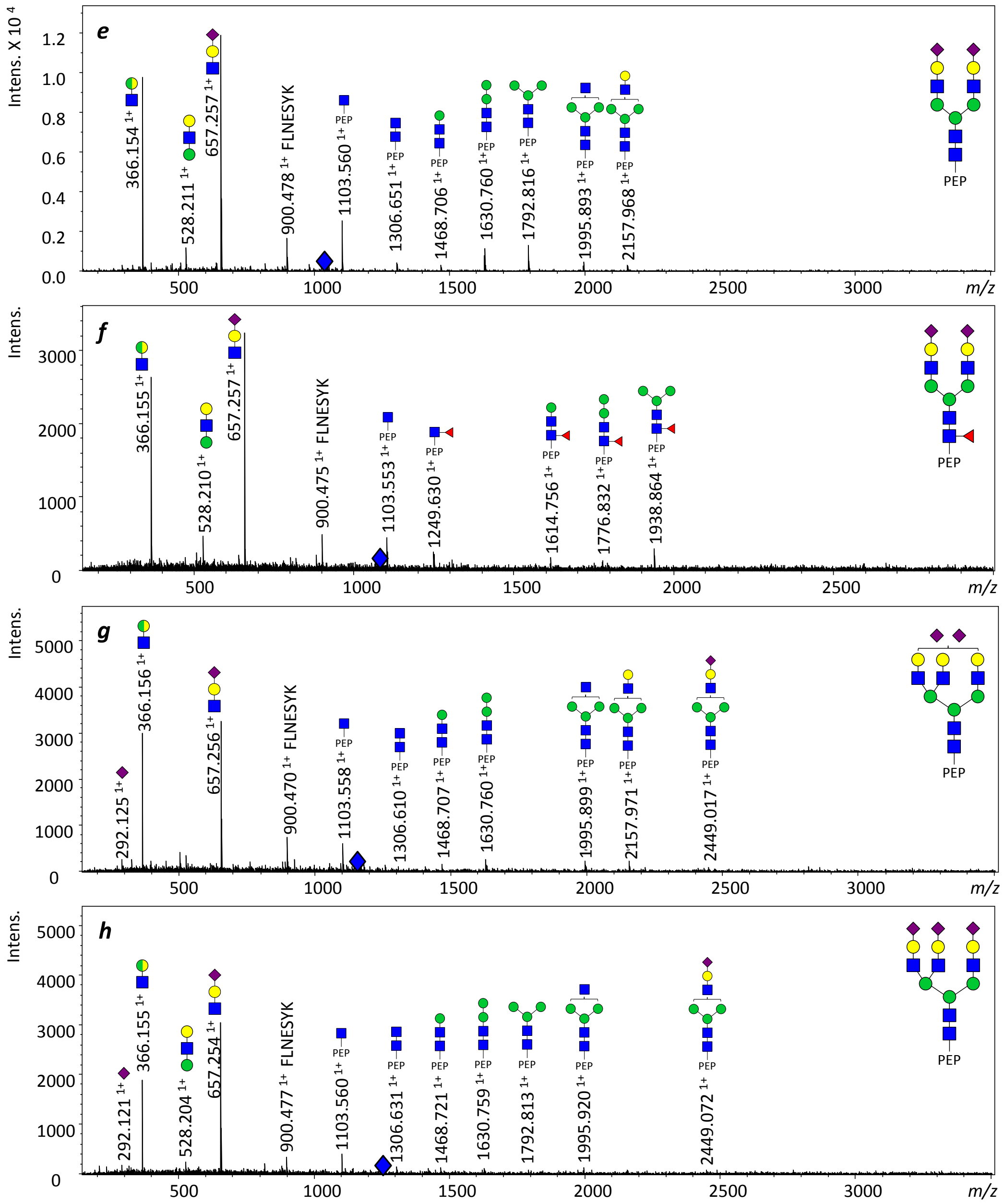

Figure S-10 (continued). The identification of seminal plasma PAP glycopeptides via tandem MS using CE-MS. (e-h) Tandem MS spectra of glycopeptides H5N4S2, H5N4F1S2, H6N5S2 and H6N5S3 on glycosylation site N_{94} with peptide backbone FLNESYK. Blue diamond marks the precursor ion. H: hexose. N: *N*-acetylglucosamine. F: fucose. S: *N*-acetylneuraminic acid (Neu5Ac). The assignment of glycan structures is based on tandem MS spectra.

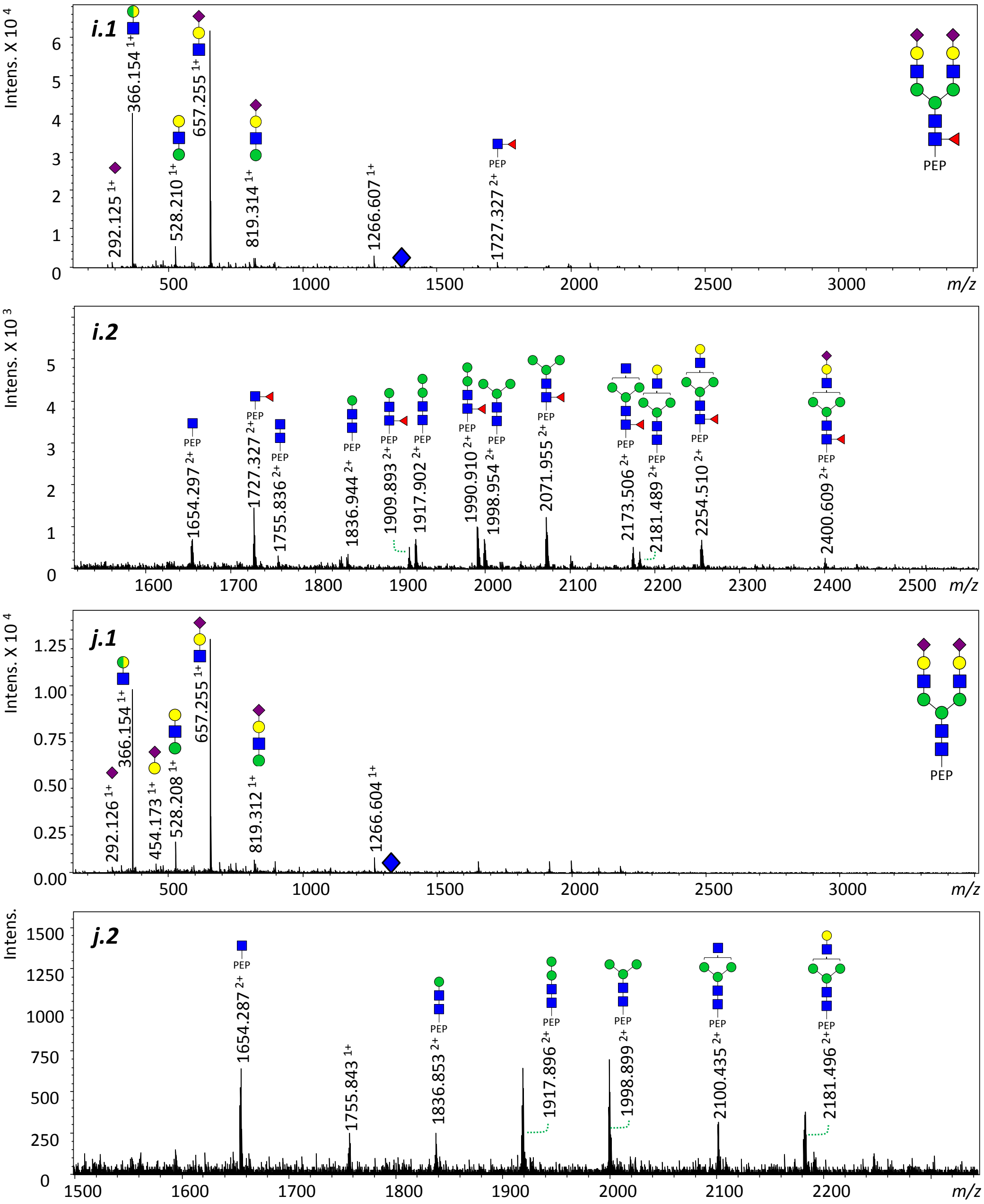

Figure S-10 (continued). The identification of seminal plasma PAP glycopeptides via tandem MS using CE-MS. (i, j) Tandem MS spectra of glycopeptides H5N4F1S2 and H5N4S2 on glycosylation site N_{220} with peptide backbone VYDPLYCESVHNFTLPSWATEDTMTK. **(i.2, j.2)** Magnification of the higher mass range of **(i.1, j.1)**. Blue diamond marks the precursor ion. H: hexose. N: *N*-acetylglucosamine. F: fucose. S: *N*-acetylneuraminic acid (Neu5Ac). Pep: peptide backbone. The assignment of glycan structures is based on tandem MS spectra.

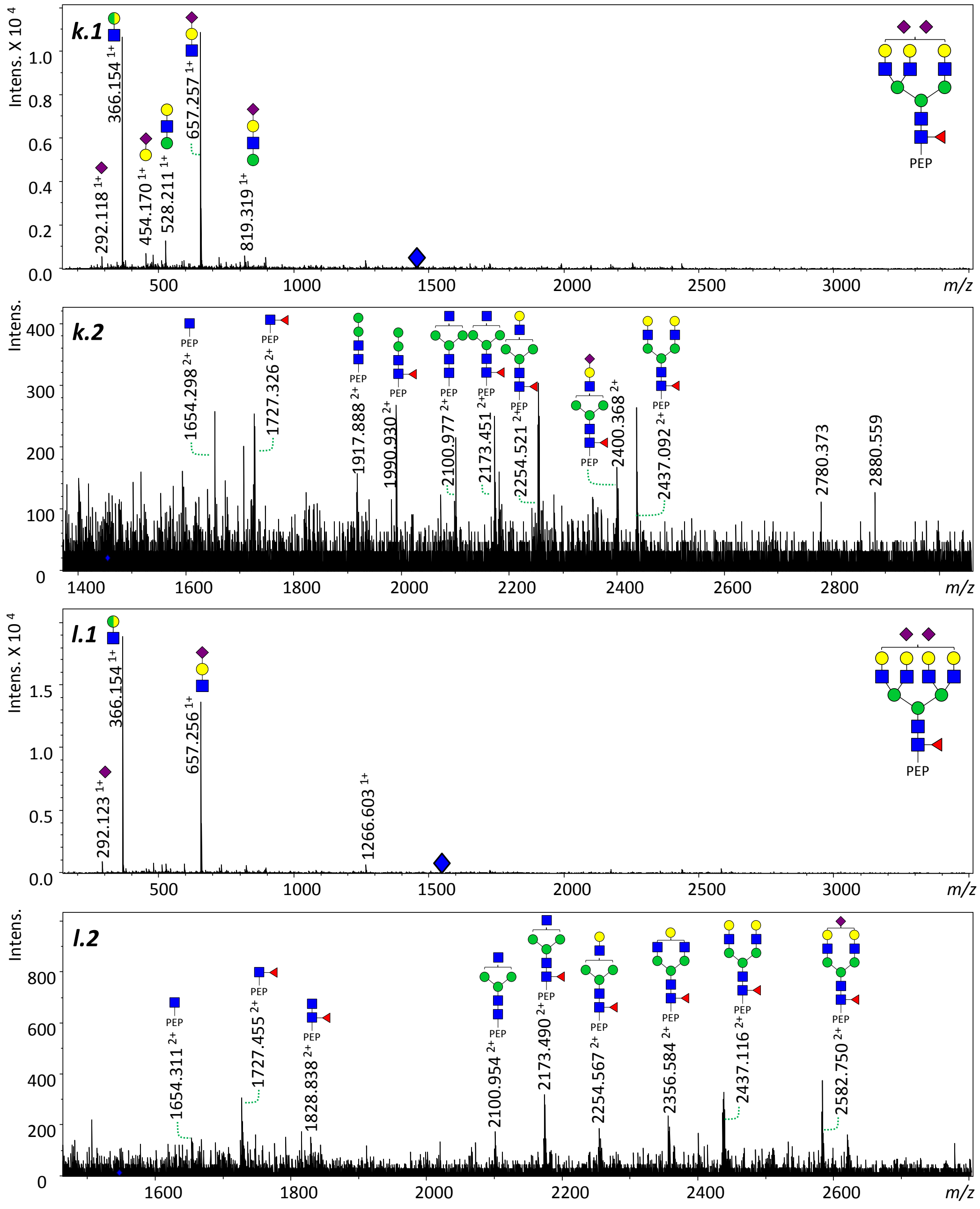

Figure S-10 (continued). The identification of seminal plasma PAP glycopeptides via tandem MS using CE-MS. (k, l) Tandem MS spectra of glycopeptides H6N5F1S2 and H7N6F1S2 on glycosylation site N_{220} with peptide backbone VYDPLYCESVHNFTLPSWATEDTMTK. **(k.2, l.2)** Magnification of the high mass range of **(k.1, l.1).** Blue diamond marks the precursor ion. H: hexose. N: *N*-acetylglucosamine. F: fucose. S: *N*-acetylneuraminic acid (Neu5Ac). Pep: peptide backbone. The assignment of glycan structures is based on tandem MS spectra.

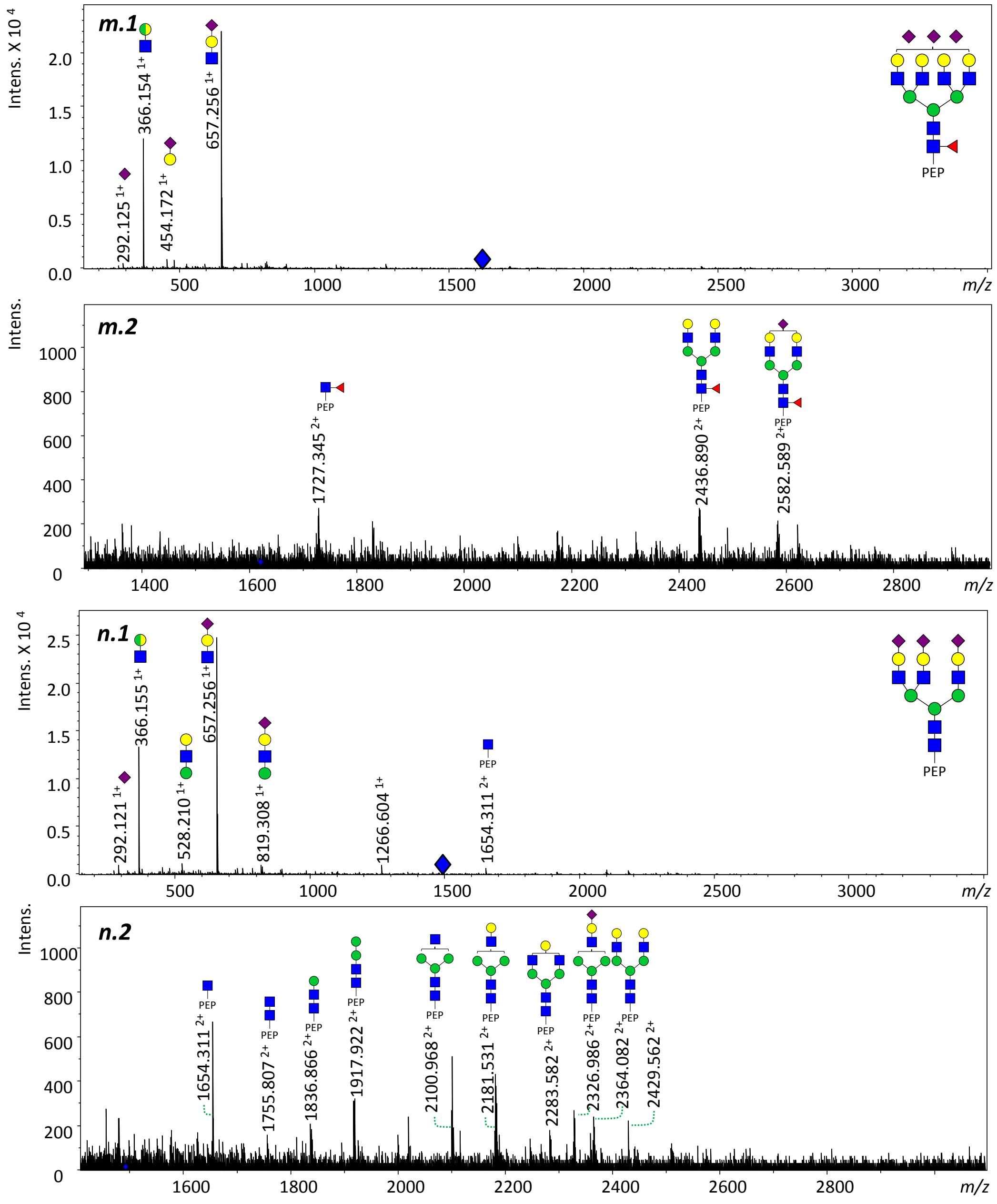

Figure S-10 (continued). The identification of seminal plasma PAP glycopeptides via tandem MS using CE-MS. (m, n) Tandem MS spectra of glycopeptides H7N6F1S3 and H6N5S3 on glycosylation site N_{220} with peptide backbone VYDPLYCESVHNFTLPSWATEDTMTK. **(m.2, n.2)** Magnification of the higher mass range of **(m.1, n.1).** Blue diamond marks the precursor ion. H: hexose. N: *N*-acetylglucosamine. F: fucose. S: *N*-acetylneuraminic acid (Neu5Ac). Pep: peptide backbone. The assignment of glycan structures is based on tandem MS spectra.

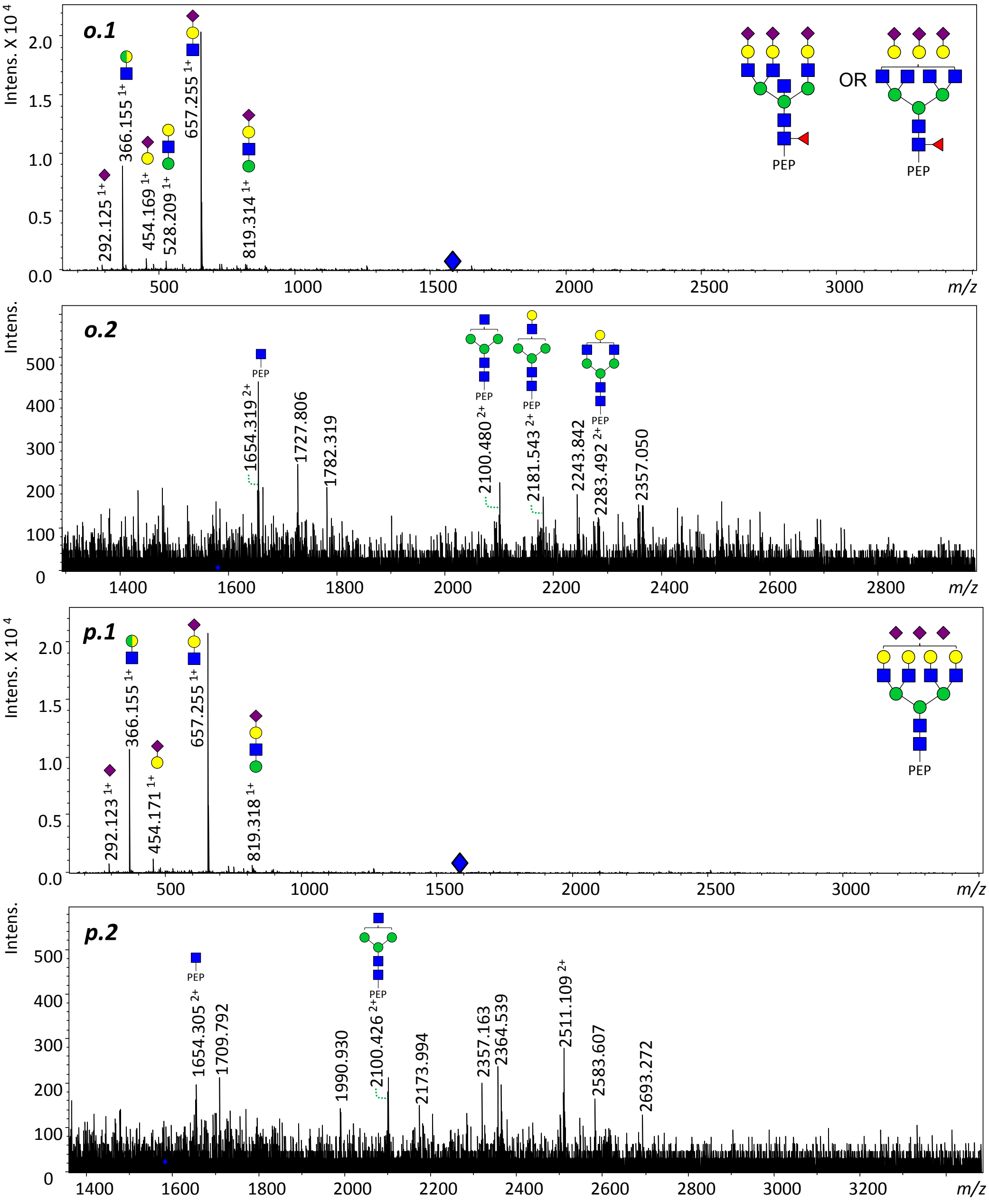

Figure S-10 (continued). The identification of seminal plasma PAP glycopeptides via tandem MS using CE-MS. (o, p) Tandem MS spectra of glycopeptides H6N6F1S3 and H7N6S3 on glycosylation site N_{220} with peptide backbone VYDPLYCESVHNFTLPSWATEDTMTK. **(o.2, p.2)** Magnification of the higher mass range of **(o.1, p.1).** Blue diamond marks the precursor ion. H: hexose. N: *N*-acetylglucosamine. F: fucose. S: *N*-acetylneuraminic acid (Neu5Ac). Pep: peptide backbone. The assignment of glycan structures is based on tandem MS spectra.

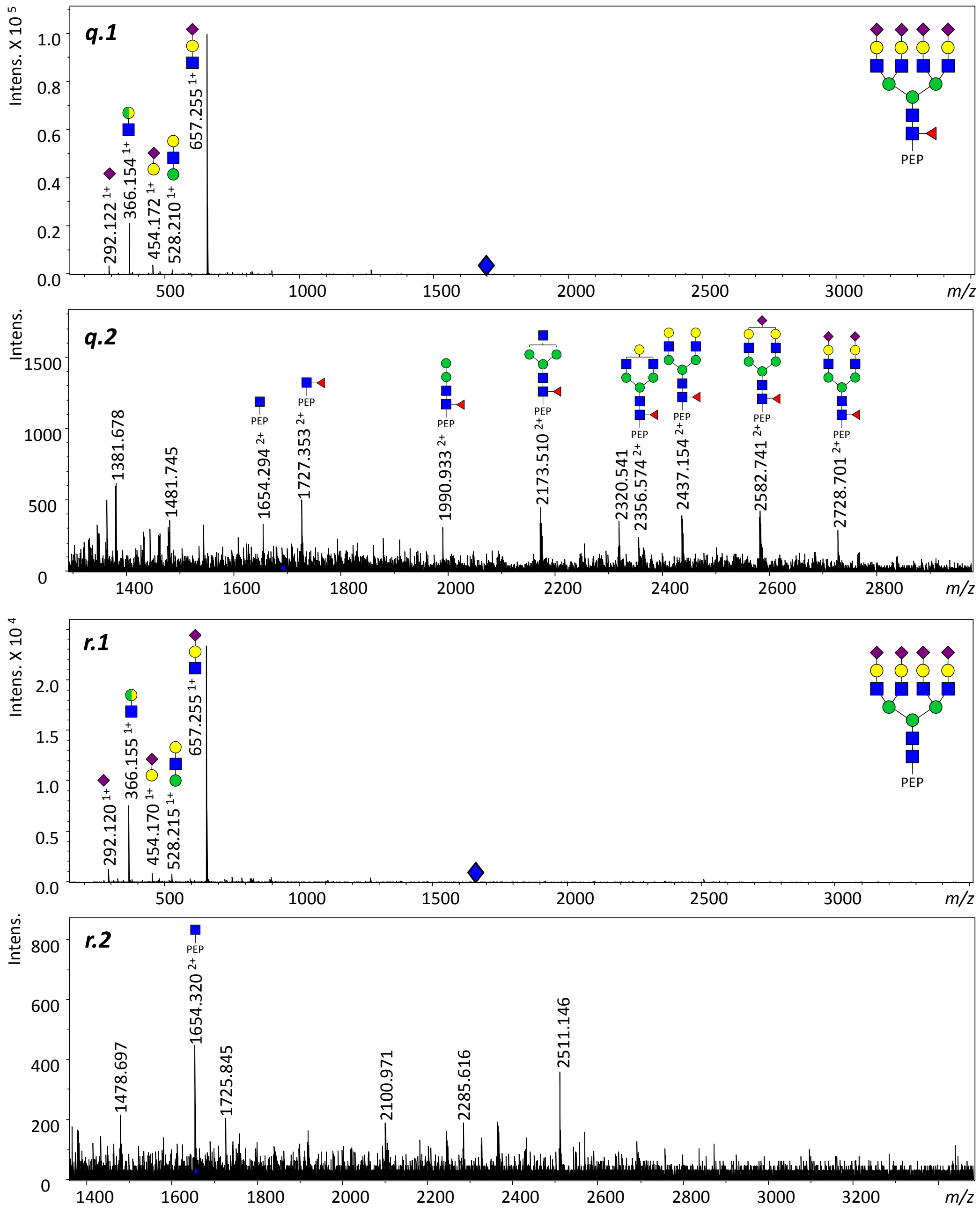

Figure S-10 (continued). The identification of seminal plasma PAP glycopeptides via tandem MS using CE-MS. (q, r) Tandem MS spectra of glycopeptides 7N6F1S4 and H7N6S4 on glycosylation site N_{220} with peptide backbone VYDPLYCESVHNFTLPSWATEDTMTK. **(q.2, r.2)** Magnification of the higher mass range of **(q.1, r.1).** Blue diamond marks the precursor ion. H: hexose. N: *N*-acetylglucosamine. F: fucose. S: *N*-acetylneuraminic acid (Neu5Ac). Pep: peptide backbone. The assignment of glycan structures is based on tandem MS spectra.

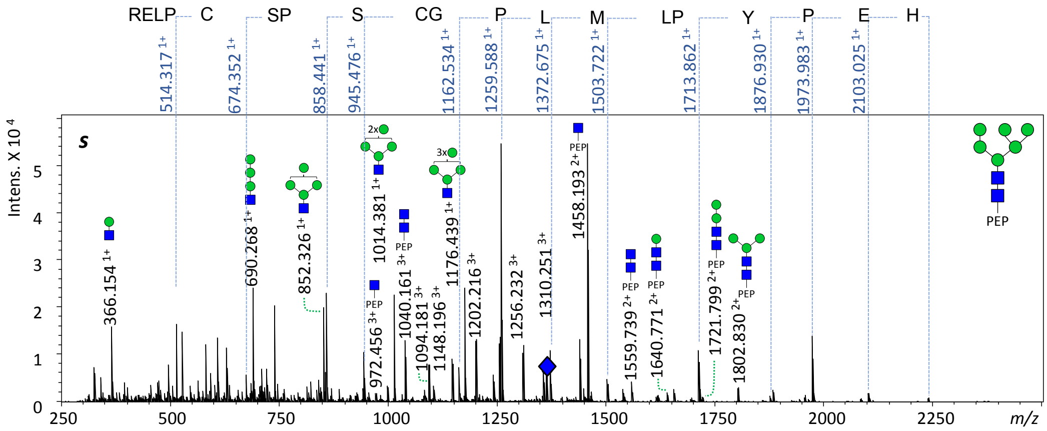

Figure S-10 (continued). The identification of seminal plasma PAP glycopeptides via tandem MS using CE-MS. (s) Tandem MS spectrum of glycopeptides H6N2 on glycosylation site N_{333} with peptide backbone NETQHEPYPLMLPGCSPSCPLER. Blue diamond marks the precursor ion. H: hexose. N: *N*-acetylglucosamine. Pep: peptide backbone. The assignment of glycan structures is based on tandem MS spectra.

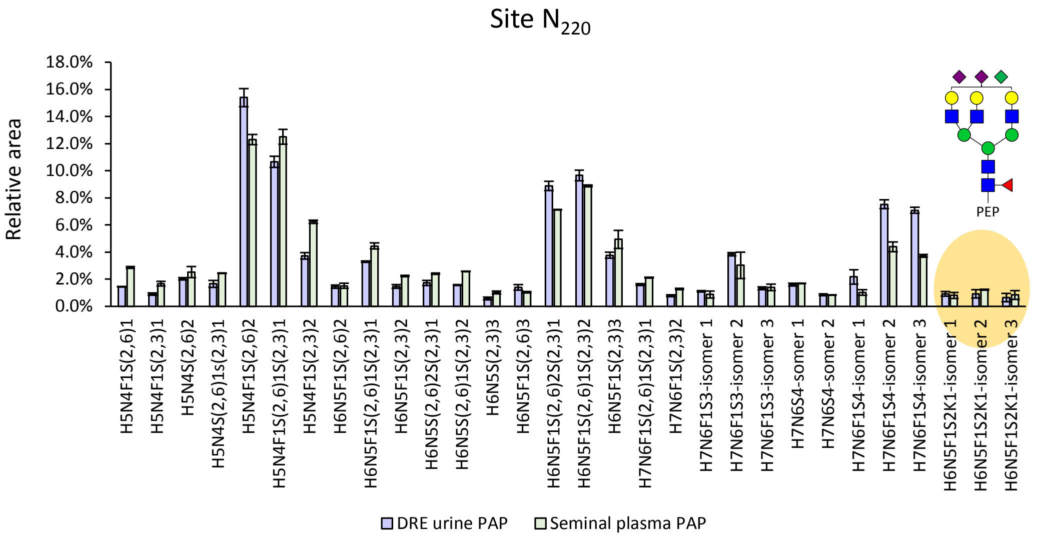

Figure S-11. Relative abundance of observed Kdn-containing glycopeptide H6N5F1S2K1-VYDPLYCESVHNFTLPSWATEDTMTK glycopeptides on glycosylation site N_{220} of PAP derived from DRE urine and seminal plasma. H: hexose. N: *N*-acetylglucosamine. F: fucose. S: *N*-acetylneuraminic acid (Neu5Ac). K: ketodeoxynononic acid (Kdn). Pep: peptide backbone. The assignment of glycan structure is based on tandem MS spectra.

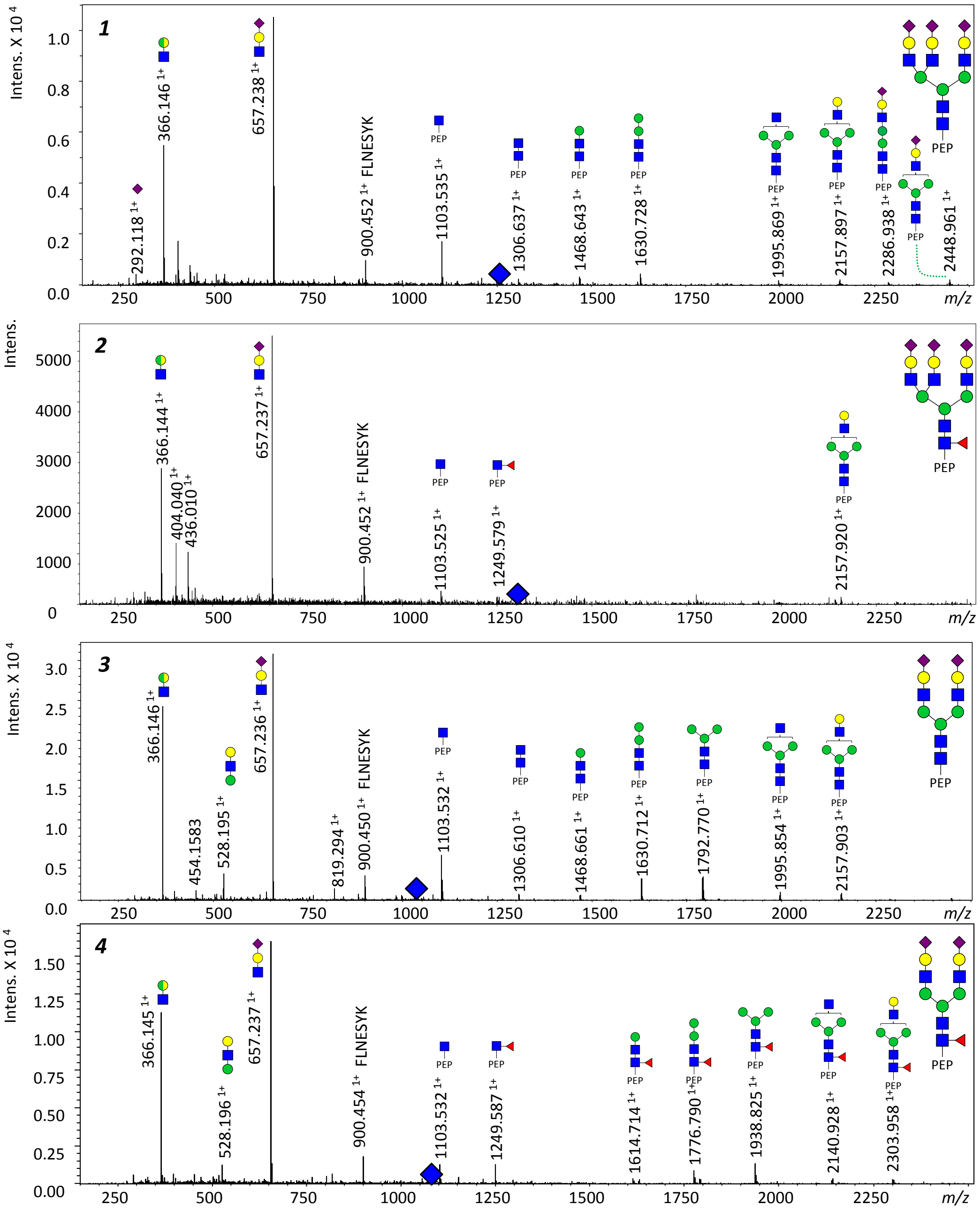

Figure S-12. The identification of DRE urinary PAP glycopeptides via tandem MS using CE-MS. (1-4) Tandem MS spectra of glycopeptides H6N5S3, H6N5F1S3, H5N4S2 and H5N4F1S2 on glycosylation site N_{94} with peptide backbone FLNESYK. Blue diamond marks the precursor ion. H: hexose. N: *N*-acetylglucosamine. F: fucose. S: *N*-acetylneuraminic acid (Neu5Ac). The assignment of glycan structures is based on tandem MS spectra.

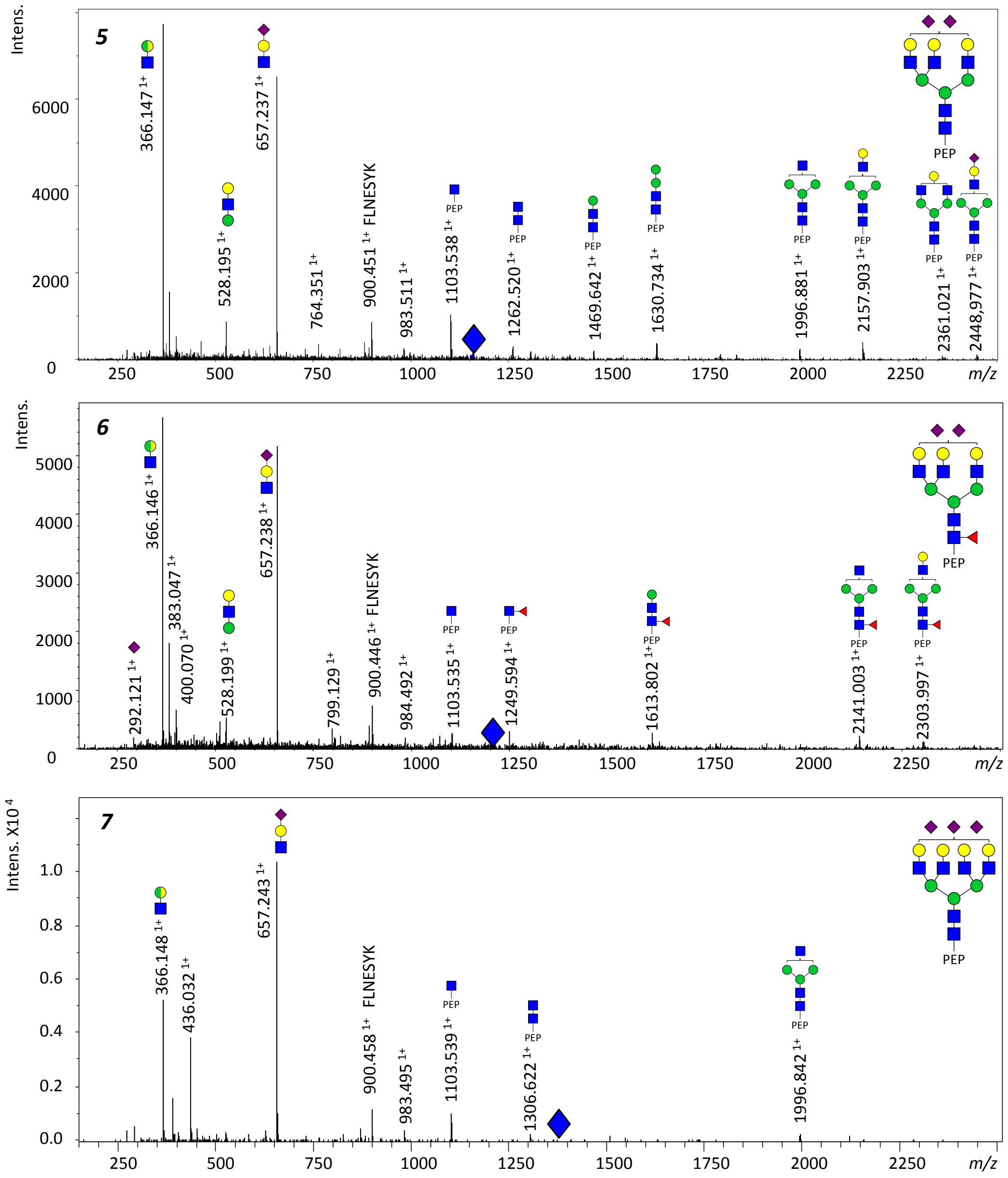

Figure S-12 (continued). The identification of DRE urinary PAP glycopeptides via tandem MS using CE-MS. (5-7) Tandem MS spectra of glycopeptides H6N5S2, H6N5F1S2 and H7N6S3 on glycosylation site N_{94} with peptide backbone FLNESYK. Blue diamond marks the precursor ion. H: hexose. N: *N*-acetylglucosamine. F: fucose. S: *N*-acetylneuraminic acid (Neu5Ac). The assignment of glycan structures is based on tandem MS spectra.

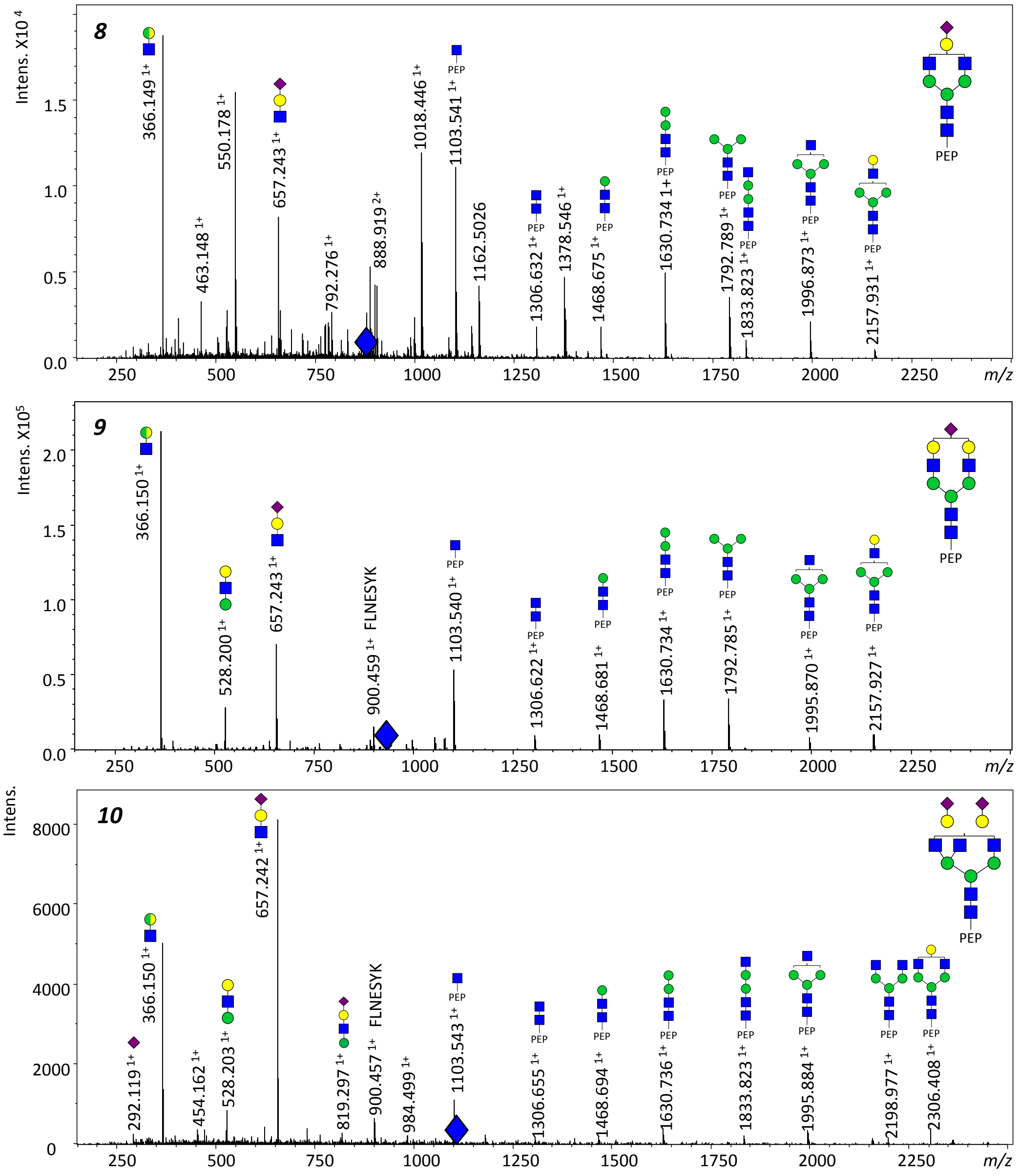

Figure S-12 (continued). The identification of DRE urinary PAP glycopeptides via tandem MS using CE-MS. (8-10) Tandem MS spectra of glycopeptides H4N4S1, H5N4S1 and H5N5S2 on glycosylation site N_{94} with peptide backbone FLNESYK. Blue diamond marks the precursor ion. H: hexose. N: *N*-acetylglucosamine. F: fucose. S: *N*-acetylneuraminic acid (Neu5Ac). The assignment of glycan structures is based on tandem MS spectra.

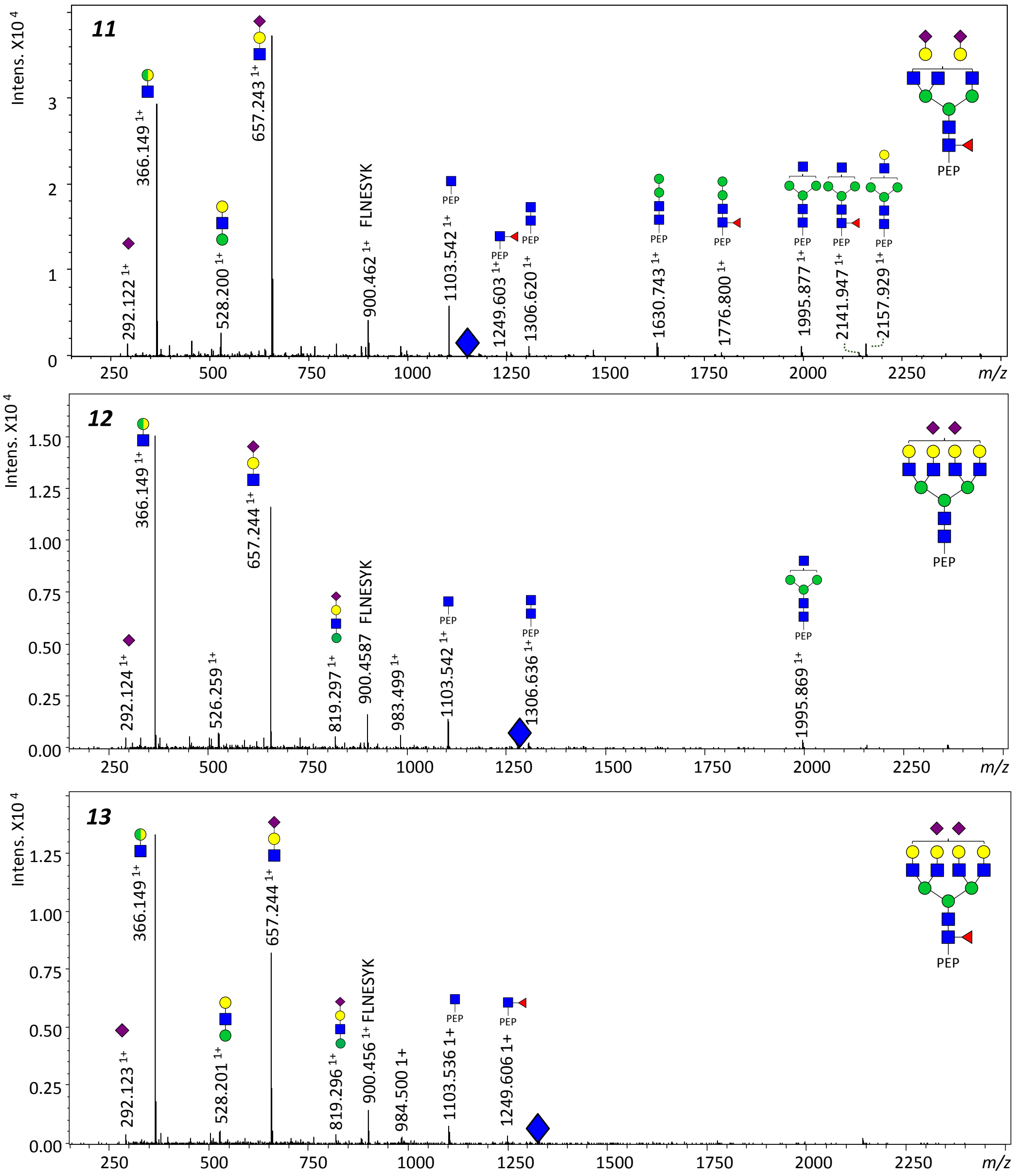

Figure S-12 (continued). The identification of DRE urinary PAP glycopeptides via tandem MS using CE-MS. (11-13) Tandem MS spectra of glycopeptides H5N5F1S2, H7N6S2 and H7N6F1S2 on glycosylation site N_{94} with peptide backbone FLNESYK. Blue diamond marks the precursor ion. H: hexose. N: *N*-acetylglucosamine. F: fucose. S: *N*-acetylneuraminic acid (Neu5Ac). The assignment of glycan structures is based on tandem MS spectra.

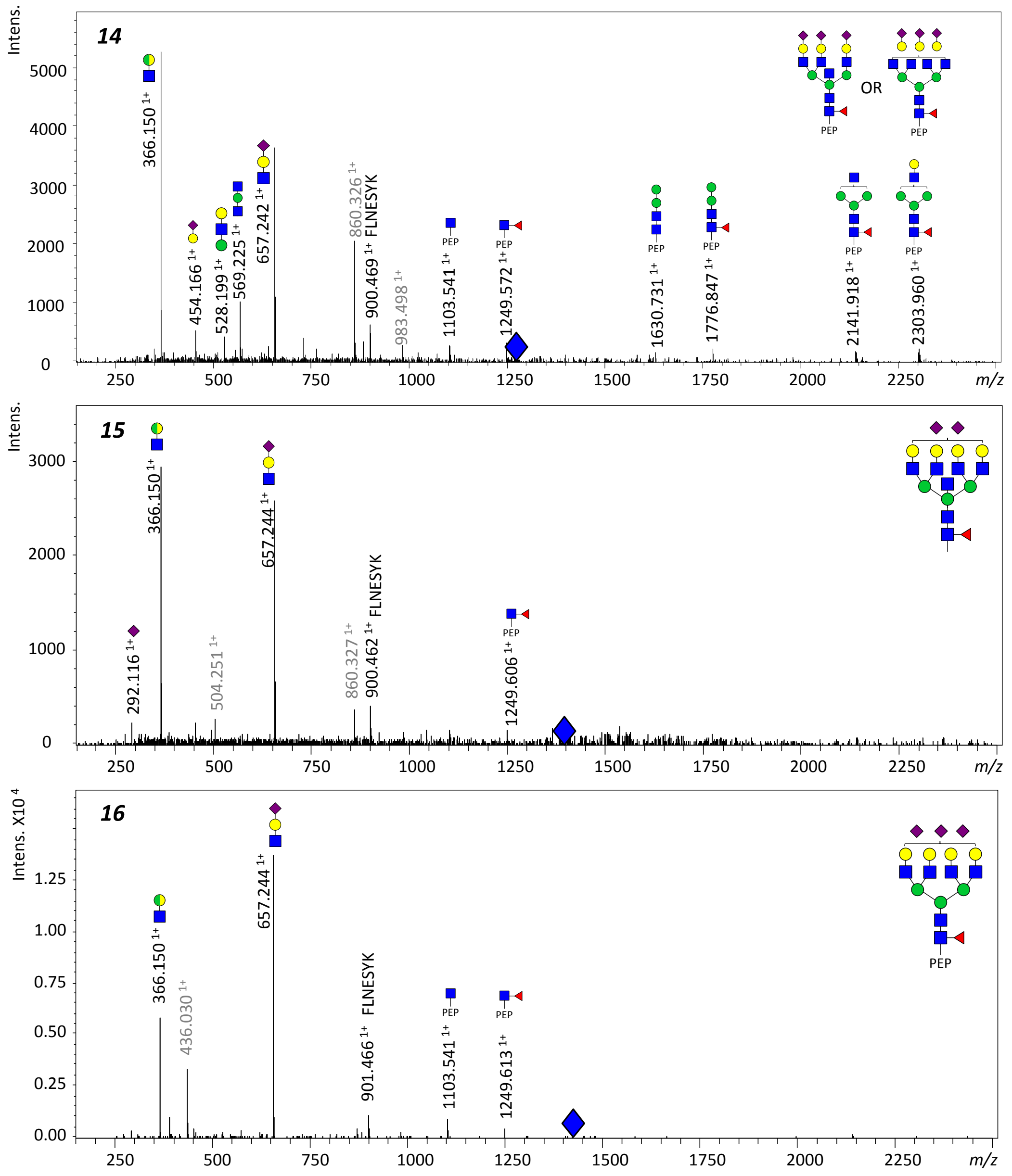

Figure S-12 (continued). The identification of DRE urinary PAP glycopeptides via tandem MS using CE-MS. (14-16) Tandem MS spectra of glycopeptides H6N6F1S3, H7N7F1S2 and H7N6F1S3 on glycosylation site N_{94} with peptide backbone FLNESYK. Fragment ions shown in grey are not identified. Blue diamond marks the precursor ion. H: hexose. N: *N*-acetylglucosamine. F: fucose. S: *N*-acetylneuraminic acid (Neu5Ac). The assignment of glycan structures is based on tandem MS spectra.

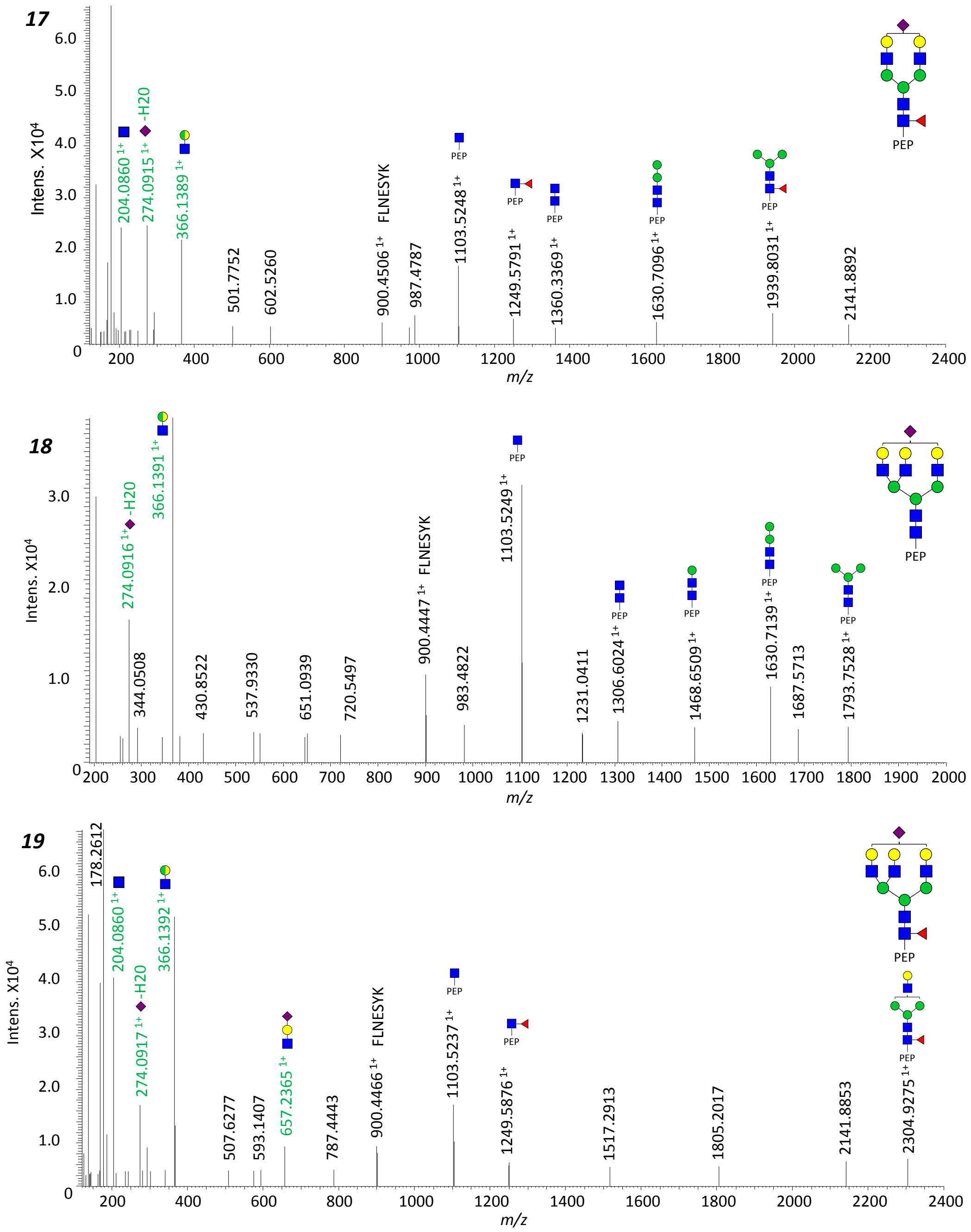

Figure S-12 (continued). The identification of DRE urinary PAP glycopeptides via tandem MS using LC-MS (Orbitrap). (17-19) Tandem MS spectra of glycopeptides H5N4F1S1, H6N5S1 and H6N5F1S1 on glycosylation site N_{94} with peptide backbone FLNESYK. Oxonium (B-)ions are highlighted in green. H: hexose. N: *N*-acetylglucosamine. F: fucose. S: *N*-acetylneuraminic acid (Neu5Ac). The assignment of glycan structures is based on tandem MS spectra.

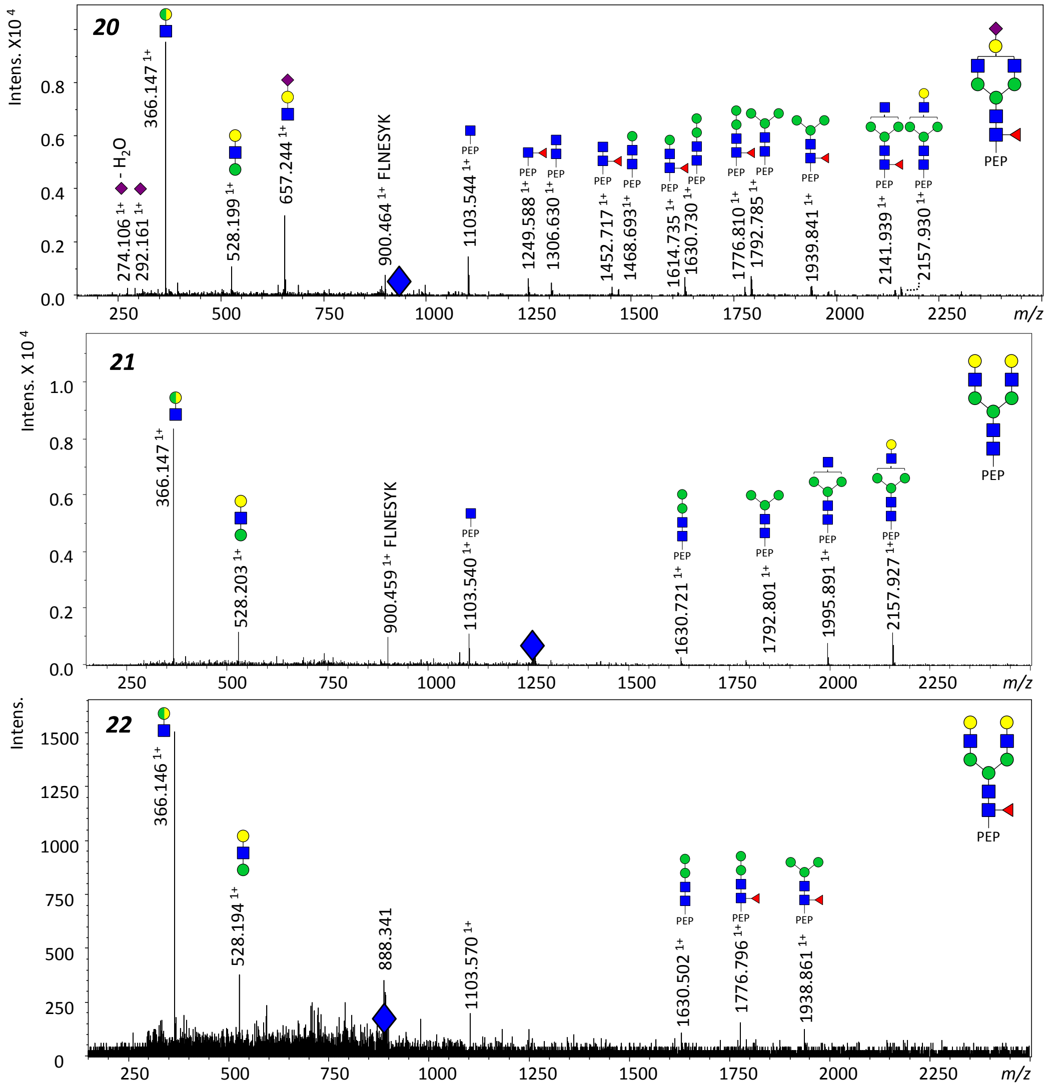

Figure S-12 (continued). The identification of DRE urinary PAP glycopeptides via targeted tandem MS using CE-MS. (20-22) Tandem MS spectra of glycopeptides H4N4F1S1, H5N4 and H5N4F1 and on glycosylation site N_{94} with peptide backbone FLNESYK. Blue diamond marks the precursor ion. H: hexose. N: *N*-acetylglucosamine. F: fucose. S: *N*-acetylneuraminic acid (Neu5Ac). The assignment of glycan structures is based on tandem MS spectra.

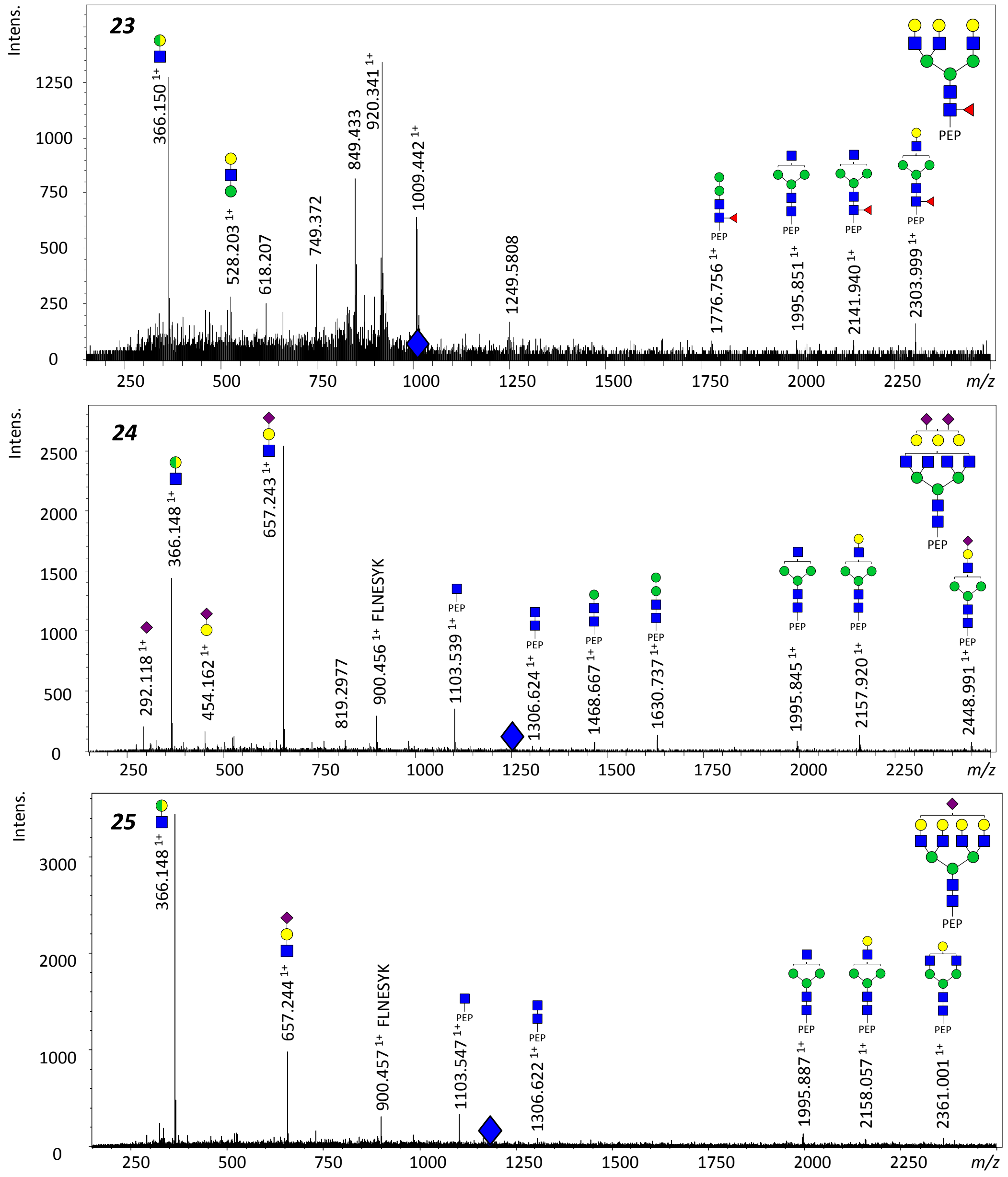

Figure S-12 (continued). The identification of DRE urinary PAP glycopeptides via targeted tandem MS using CE-MS. (23-25) Tandem MS spectra of glycopeptides H6N5F1, H6N6S2 and H7N6S1 and on glycosylation site N_{94} with peptide backbone FLNESYK. Blue diamond marks the precursor ion. H: hexose. N: *N*-acetylglucosamine. F: fucose. S: *N*-acetylneuraminic acid (Neu5Ac). The assignment of glycan structures is based on tandem MS spectra.

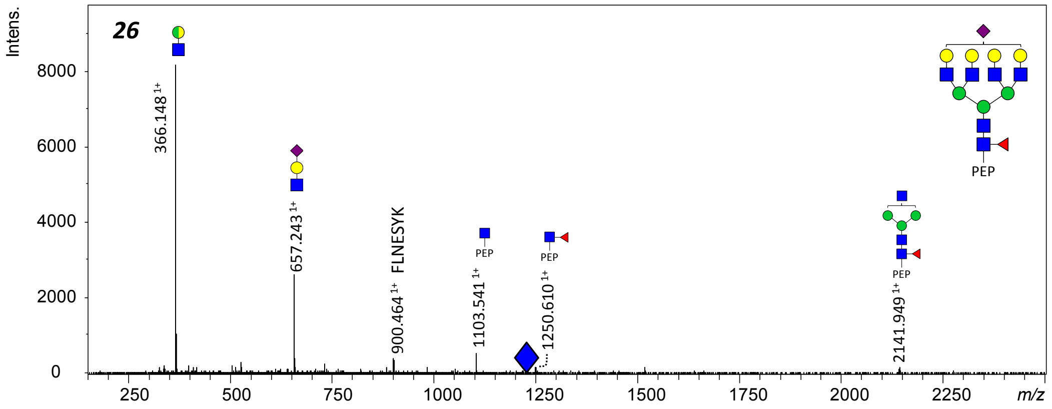

Figure S-12 (continued). The identification of DRE urinary PAP glycopeptides via targeted tandem MS using CE-MS. (26) Tandem MS spectra of glycopeptides H7N6F1S1 on glycosylation site N_{94} with peptide backbone FLNESYK. Blue diamond marks the precursor ion. H: hexose. N: *N*-acetylglucosamine. F: fucose. S: *N*-acetylneuraminic acid (Neu5Ac). The assignment of glycan structures is based on tandem MS spectra.

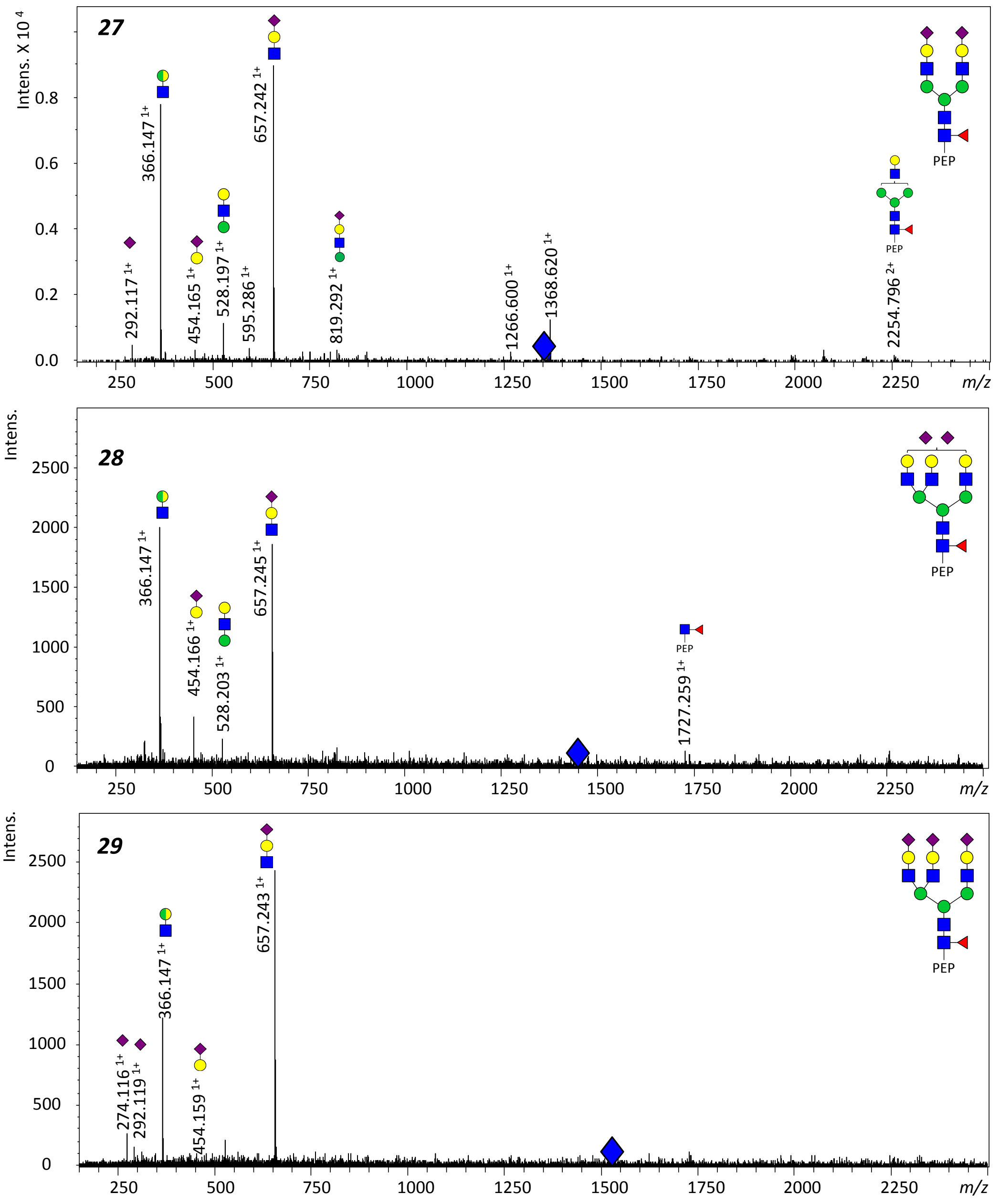

Figure S-12 (continued). The identification of DRE urinary PAP glycopeptides via targeted tandem MS using CE-MS. (27-29) Tandem MS spectra of glycopeptides H5N4F1S2, H6N5F1S2 and H6N5F1S3 and on glycosylation site N_{220} with peptide backbone VYDPLYCESVHNFTLPSWATEDTMTK. Blue diamond marks the precursor ion. H: hexose. N: *N*-acetylglucosamine. F: fucose. S: *N*-acetylneuraminic acid (Neu5Ac). The assignment of glycan structures is based on tandem MS spectra.

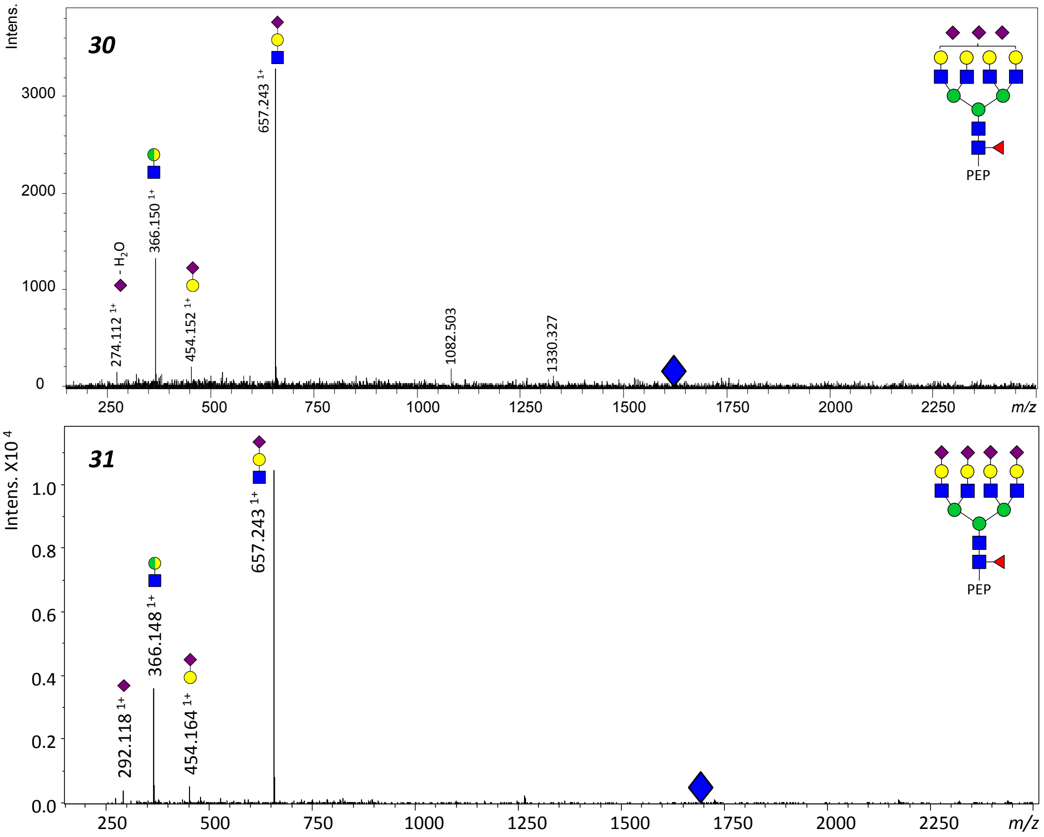

Figure S-12 (continued). The identification of DRE urinary PAP glycopeptides via targeted tandem MS using CE-MS. (30-31) Tandem MS spectra of glycopeptides H7N6F1S3 and H7N6F1S4 and on glycosylation site N_{220} with peptide backbone VYDPLYCESVHNFTLPSWATEDTMTK. Blue diamond marks the precursor ion. H: hexose. N: *N*-acetylglucosamine. F: fucose. S: *N*-acetylneuraminic acid (Neu5Ac). The assignment of glycan structures is based on tandem MS spectra.

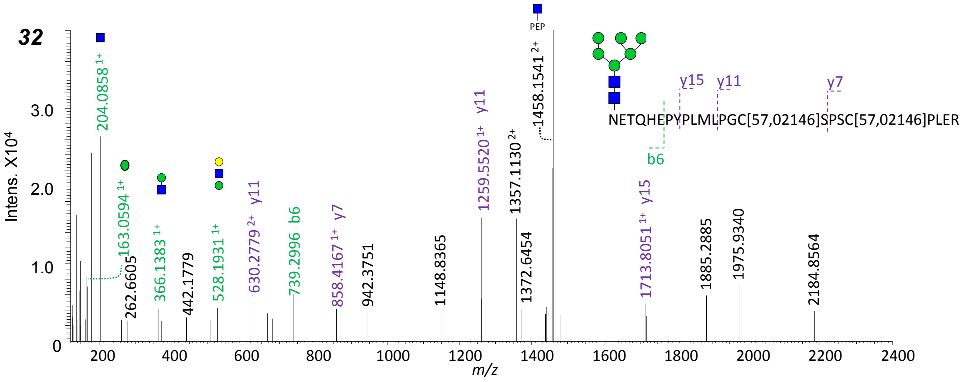

Figure S-12 (continued). The identification of DRE urinary PAP glycopeptides via tandem MS using LC-MS (Orbitrap). (32) Tandem MS spectra of glycopeptides H6N2 on glycosylation site N_{333} with peptide backbone NETQHEPYPLMLPGCSPSCPLER. Oxonium (B-)ions and Y-ions are highlighted in green and purple, respectively. H: hexose. N: *N*-acetylglucosamine. F: fucose. S: *N*-acetylneuraminic acid (Neu5Ac). The assignment of glycan structures is based on tandem MS spectra.
